# Translating New Synthetic Biology Advances for Biosensing Into the Earth and Environmental Sciences

**DOI:** 10.3389/fmicb.2020.618373

**Published:** 2021-02-04

**Authors:** Ilenne Del Valle, Emily M. Fulk, Prashant Kalvapalle, Jonathan J. Silberg, Caroline A. Masiello, Lauren B. Stadler

**Affiliations:** ^1^Systems, Synthetic, and Physical Biology Graduate Program, Rice University, Houston, TX, United States; ^2^Department of BioSciences, Rice University, Houston, TX, United States; ^3^Department of Bioengineering, Rice University, Houston, TX, United States; ^4^Department of Chemical and Biomolecular Engineering, Rice University, Houston, TX, United States; ^5^Department of Earth, Environmental and Planetary Sciences, Rice University, Houston, TX, United States; ^6^Department of Chemistry, Rice University, Houston, TX, United States; ^7^Department of Civil and Environmental Engineering, Rice University, Houston, TX, United States

**Keywords:** synthetic biology, environmental microbiology, biogeochemistry, biosensor, cell-free sensors, marine, soil, wastewater

## Abstract

The rapid diversification of synthetic biology tools holds promise in making some classically hard-to-solve environmental problems tractable. Here we review longstanding problems in the Earth and environmental sciences that could be addressed using engineered microbes as micron-scale sensors (biosensors). Biosensors can offer new perspectives on open questions, including understanding microbial behaviors in heterogeneous matrices like soils, sediments, and wastewater systems, tracking cryptic element cycling in the Earth system, and establishing the dynamics of microbe-microbe, microbe-plant, and microbe-material interactions. Before these new tools can reach their potential, however, a suite of biological parts and microbial chassis appropriate for environmental conditions must be developed by the synthetic biology community. This includes diversifying sensing modules to obtain information relevant to environmental questions, creating output signals that allow dynamic reporting from hard-to-image environmental materials, and tuning these sensors so that they reliably function long enough to be useful for environmental studies. Finally, ethical questions related to the use of synthetic biosensors in environmental applications are discussed.

## Introduction

Microbes are exquisite miniature sensors, able to sense, integrate, and respond dynamically to a wide range of environmental conditions. Biosensors are genetically-engineered microbes, or stand-alone biological components, that sense and report on specific environmental conditions of interest. Biosensors are an alternative to traditional analytical tools for detecting and measuring environmental signals because they can convert challenging and/or expensive-to-detect signals of interest into easily detectable outputs. They can also report on microbial “experiences” at the micron-scale in complex environmental matrices (Gage et al., 2008; Pini et al., 2017), providing information not otherwise available using common analytical approaches.

**Figure 1:**
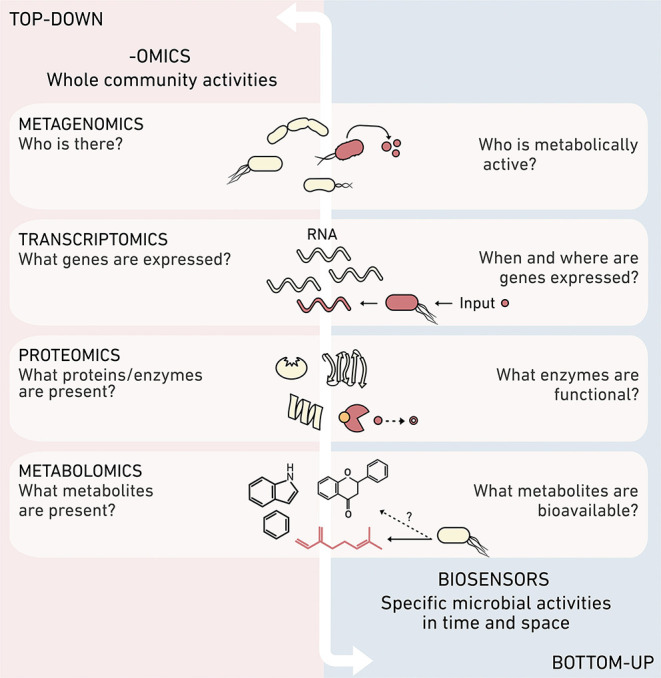
Synthetic and systems biology provide complementary information. Different -omics methods can obtain top-down systems biology data about the ensemble or organisms and biomolecules present in an environmental sample. In contrast, biosensors created using synthetic biology provide high resolution information about the reactions mediated by individual community members, such as their metabolic activities, perceived concentrations of molecules, time-dependent production or consumption of specific biomolecules, and environmental chemical processing.

Biosensors pair well with more holistic –omics approaches (Figure 1), which provide a systems-level view of the organisms, biomacromolecules, and metabolites in a sample. This -omics information can be used to reconstruct the potential of a sampled environment to perform an ecological process, for example by detecting the presence of genes that underlie the production of greenhouse gases. While -omics tools are excellent for generating ecological hypotheses (Jansson and Hofmockel, 2018), they are not always well-suited for testing individual cause-effect hypotheses, such as the dynamic roles that specific organisms and biomolecules play in greenhouse gas production. In contrast, biosensors offer a reductionist approach for testing the impact of individual cells and biomolecules on environmental outcomes. Biosensors can produce complementary spatial and temporal information on the activities of specific microbial community members, the environmental patterns that trigger cellular behaviors, and the effects of the local environment on biomolecule bioavailability.

Biosensors have been applied in environmental research (Bilal and Iqbal, 2019), but the scope of previous applications has largely focused on the detection of pollutants (van der Meer and Belkin, 2010; Pasco et al., 2011), often with the goal of informing bioremediation (Dangi et al., 2018). These biosensors have primarily used one-input, one-output systems that convert the detection of a single chemical into an easily detectable output. Here, we describe how emerging synthetic biology tools have the potential to improve the performance of traditional biosensors and expand their application to study challenging questions in the Earth and environmental sciences, including the role that cell-cell communication plays in coordinating cellular behavior in native environments, the effects of physical and chemical heterogeneity on microbial behaviors and growth, and capturing fleeting reactions like the cryptic cycling of nutrients that underlie microbial syntrophies.

**Figure 2:**
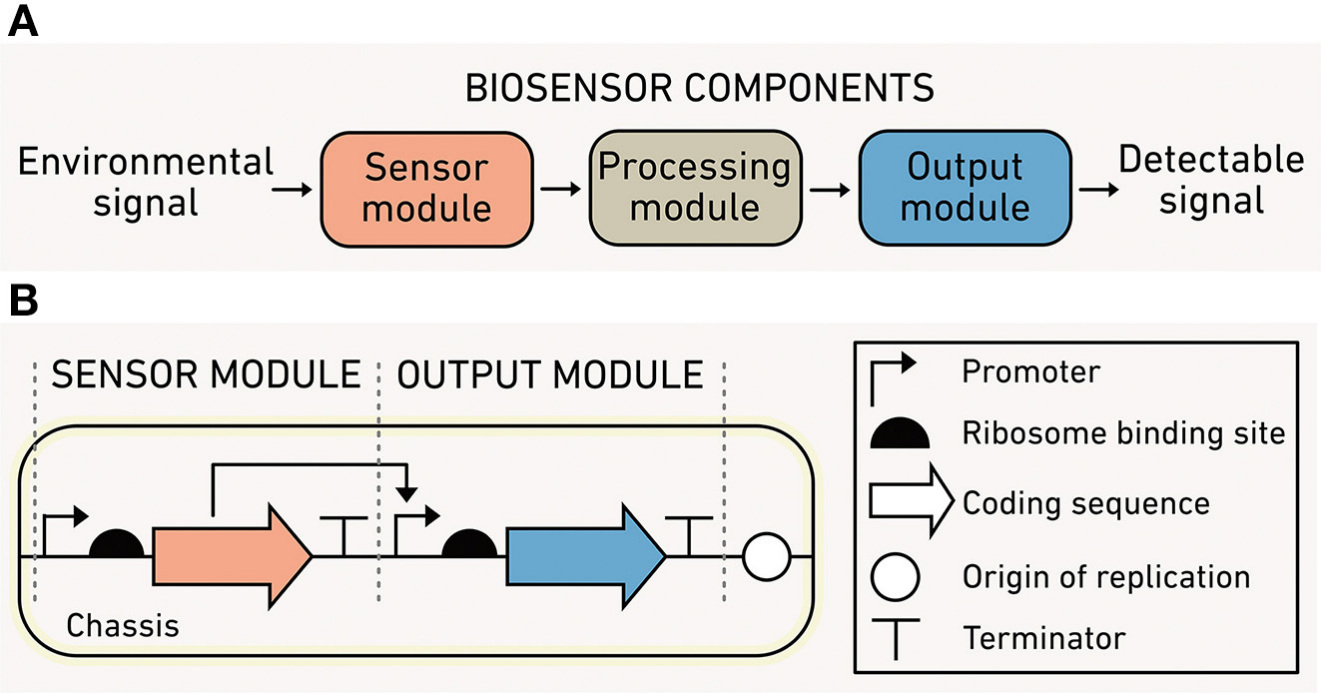
Biosensor modules and characteristics. **(A)** The sensor module (orange) converts environmental information into biochemical information, the processing module (gray) performs computations using biochemical information, and the output module (blue) translates the processed information into a detected signal. **(B)** A simple one-input, one-output biosensor illustrated using synthetic biology language.

Synthetic biology develops methods to program predictable cellular functions and includes a substantial body of work seeking to broaden biosensor capabilities. Biosensors are modular (Figure 2A) and consist of: (1) a *sensor module* that detects one or more environmental conditions as inputs, (2) a *processing module* that performs calculations using the input signals, and (3) an *output module* that produces a detectable and quantifiable signal. Biosensors are depicted using DNA circuit diagrams (Figure 2B) that include information about the genes in each module, how the expression of those genes is regulated, and how module components interact. The relationship between biosensor output and input typically has a sigmoidal shape. The dynamic range of a sensor is the difference between the threshold environmental input needed for activation, defined as the limit of detection, and the maximum input before saturation.

The recent surge of new synthetic biology modules has considerably broadened the range of inputs that can be sensed (Tables 1–3), the complexity and capabilities of processing modules, and the diversity of conditions where outputs can be detected. In turn, we are now poised to create biosensors for studying many fundamental questions in the fields of biogeochemistry, ecosystem ecology, geobiology, and environmental engineering. While a few early tools were employed within environmental samples (DeAngelis et al., 2005, 2007), these tools have not been widely accessible to the Earth and environmental science communities.

**Table 1:** Modules available for sensing intermediates in the C, N, S, and P cycles.

**Analyte sensed**		**Name**	**Mechanism**	**Mode of prior use**	**Application**	**References**
C	CO	CooA	TR	*R. rubrum*	N/A	Roberts et al., 2001
		RcoM	TR	*E. coli*	N/A	Kerby et al., 2008
	CO_2_	RegAB*	TCS	*R. capsulatus*	N/A	Ganesh et al., 2020
	CH_3_OH	FlhS/EnvZ	TCS	*E. coli*	N/A	Selvamani et al., 2017
	CH_2_O	FrmR	TR	*E. coli*	N/A	Rohlhill et al., 2017
	CH_3_X	Ada	TR	*E. coli*	N/A	Moser et al., 2013
		Unknown	Unknown	*M. extorquens*	Leaves	Farhan Ul Haque et al., 2013
	Vanillin	VanR	TR	*E. coli*	N/A	Kunjapur and Prather, 2019
		QacR	TR	Cell-free; *E. coli*	N/A	de los Santos et al., 2016
	Vanillic acid	VanR	TR	*E. coli*	N/A	Meyer et al., 2019
	p-Coumaric acid	PadR	TR	*E. coli, C. glutamicum*	N/A	Siedler et al., 2017
	Protocatachuate	PcaU	TR	*P. putida*	N/A	Jha et al., 2018
	Salicylic acid	NahR	TR	*E. coli*	N/A	Meyer et al., 2019
	DHBA	PcaU	TR	*E. coli*	N/A	Meyer et al., 2019
	Arabinose	AraC	TR	*E. coli*	N/A	Meyer et al., 2019
	Cellobiose	CelR	TR	*E. coli*	N/A	Kwon et al., 2018
	Glucose	MglB	PS	*E. coli*	N/A	Deuschle et al., 2005
	Maltose	MalE	PS	*E. coli*	N/A	Kaper et al., 2008
	Xylose	XylR	TR	*E. coli*	N/A	Ribeiro et al., 2013
N	${\text{NH}}_{4}^{+}$	Km-Amt	TCS	*K. stuttgartiensis*	N/A	Pflüger et al., 2018
	${\text{NO}}_{3}^{-}$	NarXL	TCS	*E. coli*	Soil	DeAngelis et al., 2005
	${\text{NO}}_{2}^{-}$	NarXL	TCS	*E. coli*	Soil	DeAngelis et al., 2005
	NO	NorR	TR	*E. coli*	Gut epithelium	Archer et al., 2012
		NsrR	TR	*E. coli*	N/A	McKay et al., 2018
	N_2_	NtrBC*	TCS	*E. coli*	N/A	Ganesh et al., 2020
		NtcA*	TR	Cyanobacteria	N/A	Ganesh et al., 2020
	Amino acids	GFP	translation	Cell-free	Serum	Jang et al., 2017
S	H_2_S	SQR & CstR	TR	*E. coli*	N/A	Liu et al., 2019
		SqrR	TR	*R. capsulatus*	N/A	Shimizu et al., 2017
	Polysulfide (HS_n_H, n ≥ 2)	CstR	TR	*E. coli*	N/A	Liu et al., 2019
	${\text{SO}}_{4}^{2-}$	SBP & AEQ	PS	Purified protein	Serum; urine	Hamorsky et al., 2012
	S_4_${\text{O}}_{6}^{2-}$	TtrSR	TCS	*E. coli*	Mouse gut	Daeffler et al., 2017
	S_2_${\text{O}}_{3}^{2-}$	ThsSR	TCS	*E. coli*	Mouse gut	Daeffler et al., 2017
P	${\text{PO}}_{4}^{3-}$	PstS	PS	Purified protein	N/A	Solscheid et al., 2015
	ATP	Plg2	PS	Purified protein	N/A	Branchini et al., 2015
		CRISPR/aptamer	RNA cleavage	Purified protein	N/A	Peng et al., 2020

Modules listed include both natural and engineered sensors, most of which use a single transcriptional regulator (TR), two-component system (TCS), or a protein switch (PS) to convert environmental information into biochemical information. This list excludes sensors which require electrochemical components. For each sensor, the table notes whether the mode of prior use has been within a cell, cell-free system, or isolated biomolecule. Additionally, it notes whether there has been a complex sample application in soil, serum, urine, plant leaves, or animals. Most sensors have not yet been applied (N/A) in a complex setting. Asterisks (*) denotes sensors that respond to inputs indirectly, while daggers (†) indicate that the sensing mechanism is not fully characterized.

**Table 2:** Modules available for sensing metals and other environmental parameters.

**Analyte sensed**		**Name**	**Mechanism**	**Mode of prior use**	**Application**	**References**
Metal ions	Mn(II)	MntR Mn	TR	*B. subtilis*	N/A	Huang et al., 2017
		*yybP-ykoY*	RS	Purified RNA, *B. subtilis*	N/A	Martin et al., 2019
	Zn(II)	ZraSR	TCS	*E. coli*	Synthetic wastewater	Ravikumar et al., 2012a
		CoaR	TR	*Synechocystis* sp.	Soil extract	Peca et al., 2008
		SmtB	TR	Cell-free	Municipal water	Jung et al., 2020
		CzcSR	TCS	*P. putida*	Soil extract	Liu et al., 2012
		ZntR	TR	Cell-free	Serum	McNerney et al., 2019
	Ni(II)	NrsSR	TCS	*Synechocystis* sp.	Soil extract	Peca et al., 2008
		RcnR	TR	*E. coli*	N/A	Cayron et al., 2017
		CnrYXH	TR	*R. eutropha*	Soil	Tibazarwa et al., 2001
	Co(II)	RcnR	TR	*E. coli*	N/A	Cayron et al., 2017
		CnrYXH	TR	*R. eutropha*	Soil	Tibazarwa et al., 2001
	Cu(II)	CusSR	TCS	*E. coli*	N/A	Ravikumar et al., 2012a,b
		CueR	TR	*P. putida*	N/A	Li et al., 2014
	Cu(I/II)	CsoR	TR	Cell-free	Municipal water	Jung et al., 2020
	Fe(III)	BasSR	TCS	*E. coli*	N/A	Hagiwara et al., 2004
		PmrAB	TCS	*S. enterica*	N/A	Wösten et al., 2000
	Fe(II)	BqsSR	TCS	*P. aeruginosa*	N/A	Kreamer et al., 2012, 2015
	As(III)	ArsR	TR	*E. coli*	N/A	Wan et al., 2019
	Hg(II)	MerR	TR	Cell-free	N/A	Gräwe et al., 2019
	Cd(II)	CadC	TR	*E. coli*	Soil extract	Liao et al., 2006
		CadC	TR	Cell-free	N/A	Jung et al., 2020
	Pb(II)	CadC	TR	*E. coli*	Soil extract	Liao et al., 2006
		PbrR	TR	Gram-negative bacteria	Tap water, Groundwater	Bereza-Malcolm et al., 2016
	Cr(VI)	ChrB	TR	*E. coli, O. tritici*	River water	Branco et al., 2013
Environmental parameters	O_2_	FNR	TR	*E. coli*	N/A	Myers et al., 2013
		ANR	TR	*P. fluorescens*	Soil	Højberg et al., 1999
		FixJL	TCS	*E. coli*	N/A	de Philip et al., 1990
	H_2_	HoxJA	TCS	*R. eutropha*	N/A	Lenz and Friedrich, 1998
		HupUV	TCS	*R. capsulatus*	N/A	Elsen et al., 2003

*Modules listed include both natural and engineered sensors which use a single transcriptional regulator (TR), two-component system (TCS), or riboswitch (RS) to convert environmental information into biochemical information. This list excludes sensors which require electrochemical components. For each sensor, the table notes whether prior use has been within a cell, cell-free system, or isolated biomolecule. Additionally, it notes whether there has been a complex sample application in soil, sediment, wastewater, or serum. Most sensors have not yet been applied (N/A) in a complex environmental setting*.

**Table 3:** Modules available for osmolytes, cell-cell signals, and other common environmental parameters.

**Analyte sensed**		**Name**	**Mechanism**	**Mode of prior use**	**Application**	**References**
Osmolytes	Osmotic stress	*proU* promoter	TR	*P. putida, P agglomerans*	Soil	Herron et al., 2010
	Trimethylamine N-oxide	TorTSR	TCS	*E. coli*	N/A	Schmidl et al., 2019
	Ectoine	AraC-ect	TR	*E. coli*	N/A	Chen et al., 2015
	Glutamate	DegS-EnvZ	TCS	*E. coli*	N/A	Ravikumar et al., 2018
	Glycine betaine	CFP, GBP, & YFP	PS	*E. coli*	N/A	Ahmad et al., 2016
Cell-cell signals	C6 AHL	LuxR	TR	*E. coli*	Soil	Cheng et al., 2018
		ScbR	TR	Cell-free	N/A	Yang et al., 2009
	C12 AHL	LasR	TR	*E. coli*	Soil	Cheng et al., 2018
		LasR	TR	Cell-free	Sputum	Wen et al., 2017
	Quercetin	QdoR	TR	*E. coli*	DOC	Del Valle et al., 2020
	Naringenin	FdeR	TR	*E. coli*	DOC	Del Valle et al., 2020
		TtgR	TR	Cell-free	N/A	Jung et al., 2020
	Luteolin	FdeR	TR	*E. coli*	DOC	Del Valle et al., 2020
		NodD1-FdeR	TR	*E. coli*	N/A	De Paepe et al., 2019
	Kaempferol	QdoR	TR	*E. coli*	N/A	Siedler et al., 2014
	γ-butyrolactones	SbcR	TR	Cell-free	N/A	Yang et al., 2009
	Salicylic acid	NahR	TR	*E. coli*	N/A	Meyer et al., 2019
	Ethylene	SynEtr1	TCS	*Synechocystis* sp.	N/A	Lacey and Binder, 2016
	AIP	AgrAC	TCS	*S. aureus*	N/A	Sturme et al., 2002
	GBAP	FscAC	TCS	*E. faecalis*	N/A	Sturme et al., 2002
	Phr	Rap	TR	*B. subtilis*	N/A	Bareia et al., 2018
	ComX	ComAP	TCS	*B. subtilis*	N/A	Bareia et al., 2018
Light	UV-violet	UirSR	TCS	*E. coli*	N/A	Ramakrishnan and Tabor, 2016
	Blue	YF1-FixJ	TCS	*E. coli*	N/A	Möglich et al., 2009
	Blue	Vvd	PS	*E. coli*	N/A	Sheets et al., 2020
	Green	CsaSR	TCS	*E. coli*	N/A	Tabor et al., 2011
	Red	Cph8-EnvZ	TCS	*E. coli*	N/A	Levskaya et al., 2005
pH	5–7	RstA	TCS	*E. coli*	N/A	Hoynes-O'Connor et al., 2017
	5.5–7	VirA-ChvE	TCS	*A. tumefaciens*	N/A	Gao and Lynn, 2005
	6–8	SO_4387; SO_4388	TCS	*E. coli*	N/A	Schmidl et al., 2019
Temperature	27–37°C	CspA	TR	*E. coli*	N/A	Hoynes-O'Connor et al., 2017
	32–46°C	TlpA & Tcl	TR	*E. coli*	Mouse tissue	Piraner et al., 2017
	25–40°C	TEV & CI	TR	*E. coli*	N/A	Zheng et al., 2019
	29–37°C	RNAs	RS	Cell-free	N/A	Sen et al., 2017

*Modules listed include both natural and engineered sensors which use a single transcriptional regulator (TR), two-component system (TCS), a protein switch (PS), or riboswitch (RS) to convert environmental information into biochemical information. This list excludes sensors which require electrochemical components. For each sensor, the table notes whether the mode of prior use has been within a cell, cell-free system, or isolated biomolecule. Additionally, it notes whether there has been a complex sample application in soil, dissolved organic carbon (DOC), sputum, or animals. Most sensors have not yet been applied (N/A) in a complex environmental setting*.

The purpose of this review is to discuss the direct implications of recent advances in synthetic biology for creating biosensors relevant to hard-to-study environmental problems. The intended audiences for this review are: (1) environmental scientists and engineers who would benefit from more information on the breadth of current biosensor capabilities, and (2) synthetic biologists who are developing biosensors and would benefit from understanding environmental needs. First, in section A, we discuss challenges in the study of Earth and environmental systems that biosensors are uniquely poised to overcome. We also highlight how synthetic biology can be used to build custom biosensors for use within complex systems to address these hard questions. In sections B and C, we outline the state of synthetic biology and the explosion of biological programs that enable the creation of living and cell-free biosensors. In section D, we highlight needed developments in synthetic biology to advance applications in the Earth and environmental sciences. Finally, in section E, we examine the ethical issues associated with the use of synthetic biology to study the environment.

## A. Biosensors Can Address Hard Environmental Challenges

Biosensors create unique opportunities to study ecosystems from the microbial perspective because they are continuously sensing information at the micron scale. In this section, we describe how advanced biosensors offer the opportunity to address outstanding questions of interest to environmental researchers, including the sensing of bioavailable concentrations of chemicals at micron scales, reporting on direct interactions between different organisms, and recording information in cells while preserving the spatial and temporal heterogeneity in the natural environment.

### Biosensors Can Report on Complex Microbial Interactions

Understanding how microbes interact and function collectively is crucial to predicting how communities will respond to future perturbations, since the combined actions of diverse community members drive ecological processes (Widder et al., 2016). Microbial interactions arise from direct contact between microbes and through indirect communication mediated by biomolecules (Visick and Fuqua, 2005; Prindle et al., 2015). Here, we focus on two examples where biosensors can be used to study the influence of environmental conditions on microbial interactions via: (1) cell-to-cell signaling mediated by diffusible chemical signals, and (2) horizontal gene transfer of conjugative plasmids.

#### Biosensors Can Monitor How the Environment Influences Cell-Cell Signaling

**Figure 3:**
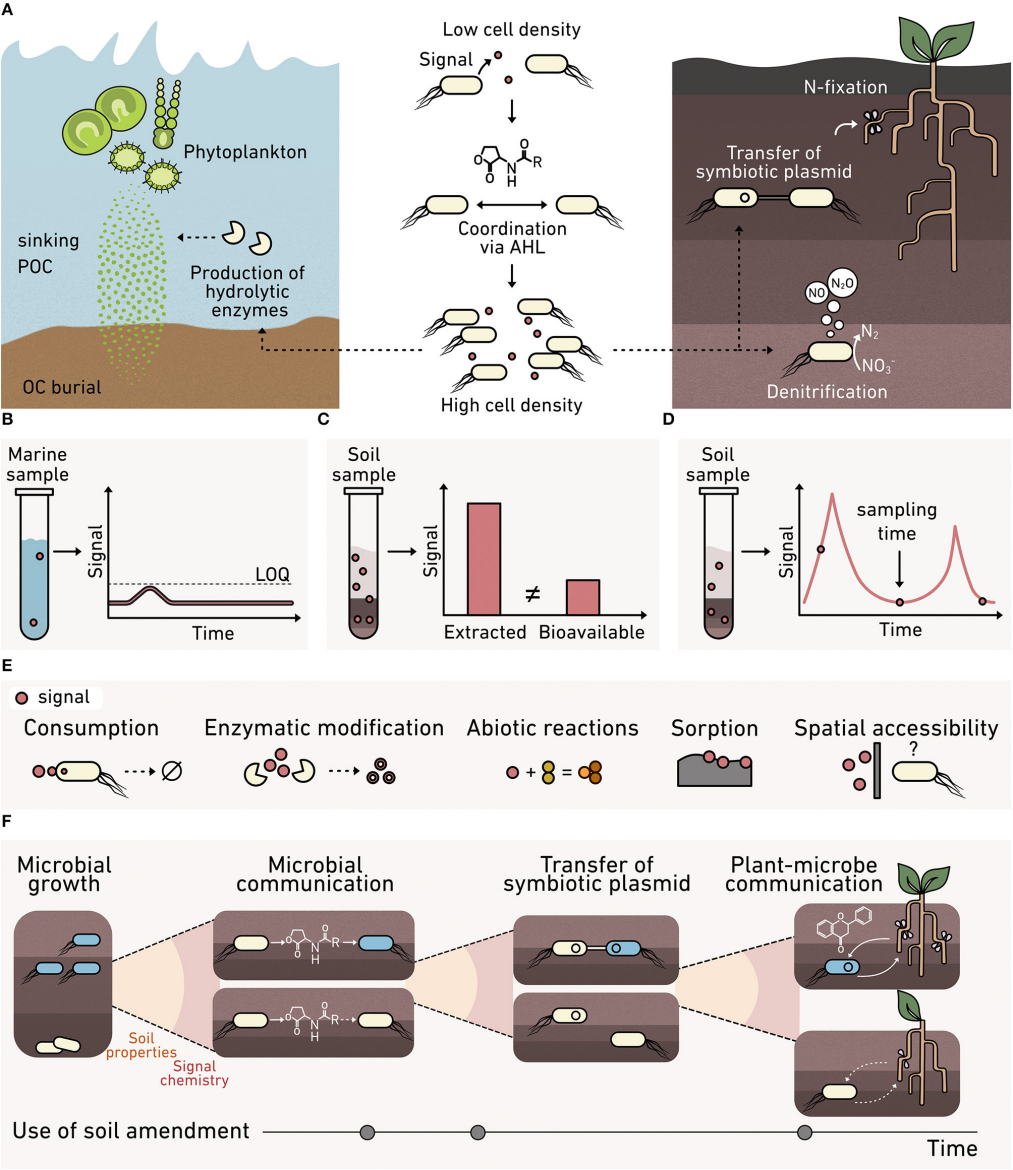
Monitoring cell-cell communication in environmental materials. **(A)** AHL cell-cell signaling (*middle*) regulates processes at the cm-scale that contribute to fluxes at a planetary scale, including: (*left*) the production of enzymes that degrade marine sinking particulate organic carbon, and (*right*) the transfer of symbiotic plasmids encoding nitrogen-fixing machinery and the production of nitrous oxide. Analytical chemistry methods for monitoring signals can present: **(B)** high limits of quantification (LOQ) that preclude signal detection, **(C)** an overestimation of signal levels, since spatial variability and bioavailability are not reflected in bulk extraction data, and **(D)** limited temporal insight because samples are consumed during analysis. **(E)** Biosensors can provide a microbe's perspective on the bioavailability of a signal at the micron scale, which varies due to consumption, enzymatic modification, abiotic chemical modification, sorption into the organo-mineral phase, and spatial accessibility. **(F)** Multiple biosensors are needed to understand the complex signaling that underlies plant-microbe symbiosis whose formation is critical to crop productivity. Biosensors are needed to understand how soil properties, signal chemistry, and amendments affect: (1) microbial growth, (2) microbe-microbe AHL signaling that underlies symbiotic plasmid transfer, and (3) plant-microbe communication mediated by flavonoids and nodulation factors that cause symbiosis formation.

Bacteria use the accumulation of diffusible biomolecular signals to regulate community behaviors through a process called quorum sensing (Figure 3A), which contributes to carbon and nitrogen cycling at the planetary scale (Hmelo, 2017). One common signal family is N-acyl homoserine lactones (AHLs). These signals are used by marine bacteria to regulate the production of enzymes that decompose sinking particulate organic carbon (POC) (Krupke et al., 2016). Sinking POC dynamics play a key role in the global carbon cycle, driving the transfer of carbon from the atmosphere to the ocean and seafloor (Krupke et al., 2016), and AHL controls in this process remain poorly understood.

AHLs also modulate nitrogen cycling in terrestrial systems by regulating denitrification and the transfer of nitrogen-fixing machinery among bacteria (Toyofuku et al., 2007; Aminov, 2011). Currently, it is unclear how soil properties, such as structure, water potential, and mineralogy, affect AHL signaling. Insight is needed into the roles that soils play in modulating AHL signaling to inform agronomic practices that support N-fixing plant-microbe symbiosis; this knowledge could minimize the need for fertilizer application. Furthermore, AHLs are used by microbial communities harnessed in engineered systems, such as biofilms and flocs present within wastewater treatment plants (Huang et al., 2016). To control biofilm formation and selectively enrich beneficial microbes for water treatment, while deselecting for bacteria that cause biofouling, there is a need to understand AHL regulation of biofilm assembly and how changing water treatment conditions affect AHL signaling *in situ*.

To understand how diffusible signals mediate ecosystem processes, it is critical to establish when these diffusible signals reach a threshold concentration necessary to activate gene expression across natural environmental materials, which vary spatially and dynamically in composition. For this reason, it is critical to measure the “bioavailable” fraction of signals, defined as the concentration that a microbe experiences, rather than the total concentration in a sample. There are several challenges associated with studying bioavailability within complex environmental materials. Extremely low-level chemical concentrations (Figure 3B) can be challenging to monitor by traditional methods where the limit of detection using an analytical instrument can be several orders of magnitude higher than that required to influence a microbial behavior (Morin et al., 2003). As an example, biosensors can detect AHLs at sub nM levels, while the detection threshold for gas chromatography is in the low μM range (Gao et al., 2016).

Traditional chemical extraction methods can also introduce bias by overestimating or underestimating the bioavailable fraction (Figure 3C), depending on the method employed for measurement (Cipullo et al., 2018). Some signals are apolar, limiting their solubility in water and promoting their sorption to organic matter and inorganic solids (Thompson and Goyne, 2012). Thus, the signal levels observed following extraction can be significantly higher than the bioavailable fraction (Schmidt et al., 2011). In addition, many analytical tools require destruction of the sample, which limits throughput when analyzing an environmental process over time (Figure 3D). A key benefit of using biosensors to detect chemicals in the environment is that they report on the bioavailable fraction *in situ* (Figure 3E). The bioavailable fraction of a chemical can dynamically change in space and time due to consumption by microbes, modification by secreted enzymes, and abiotic conditions like soil water content and the presence of surfaces that may sorb or modify those chemicals.

To date, there have been limited biosensor applications to study cell-cell signaling in matrices. One study investigated the link between AHL signaling and the production of extracellular enzymes (DeAngelis et al., 2008). This study found that one AHL class regulates nitrogen mineralization in the rhizosphere, although it did not examine the full spectrum of AHLs that are made by soil microbes. AHL biosensors have also been used to study how soil amendments, such as pyrolyzed organic matter (biochar), change AHL bioavailability (Masiello et al., 2013; Gao et al., 2016). These studies found that the physicochemical properties of the microbe's soil habitat, such as biochar surface area and pH following amendment, affect AHL bioavailability. In many of these studies, biosensors have been built using easy-to-manipulate *Escherichia coli* strains that are unlikely to survive under many environmental conditions and timescales of interest (Adams, 2016).

Recent innovations in cellular outputs (see section C) are expanding the environments where biosensors can be used and the types of environmental processes that can be studied. Biosensors that produce indicator gases instead of traditional fluorescent molecules allow non-invasive measurements of AHL production and degradation rates in soils (Cheng et al., 2018). In addition, the ability to design biosensors with high specificity for different chemicals, such as peptide signals used by gram-positive bacteria and plant root exudates, offers the potential to study signaling mediated by molecules with an even wider range of physicochemical properties. These developments open up the possibility of studying how different land management decisions impact the bioavailability of signaling molecules beyond AHLs, examining microbial behaviors regulated by this larger range of biomolecules, and investigating the ecological processes controlled by a wider range of microbes.

Biosensors could be useful for connecting land management practices to cell-cell communication that underlies fixed nitrogen production. Beyond observing if nitrogen fixation is occurring, advanced biosensors could monitor how soil composition affects each step required for nodule formation in plants (Figure 3F). Newly tractable questions include identifying soil controls on (1) the growth of nitrogen-fixing symbionts, and (2) AHL-mediated communication controlling the exchange of symbiotic plasmids encoding nitrogen-fixing machinery. Beyond processes mediated by AHL signaling, it is now possible to explore: (3) flavonoid-mediated plant-to-microbe communication required for initiating symbiosis (Del Valle et al., 2020), as well as (4) nod factor mediated microbe-to-plant communication critical to nodule formation (Wang et al., 2018). In the future, detailed insight into different signaling processes will be crucial to understanding how land use practices can maximize biological fixation of nitrogen and minimize fertilizer application.

### Biosensors Can Quantify How Horizontal Gene Transfer (HGT) Varies With Environment

**Figure 4:**
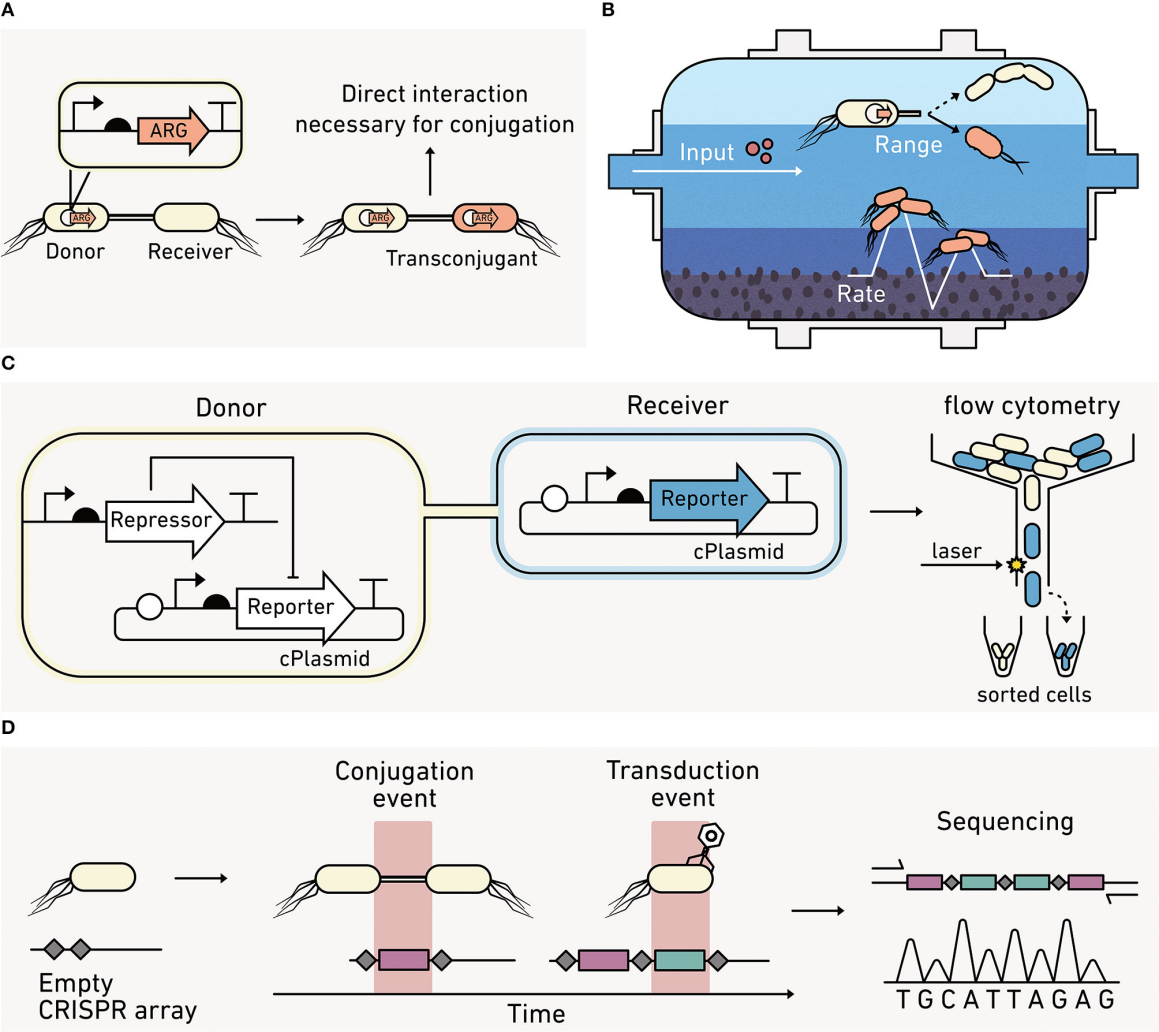
Biosensors can report on horizontal gene transfer (HGT) between cells. **(A)** Cells use HGT of antibiotic resistance genes (ARG) to improve their fitness. **(B)** In engineered systems, like wastewater treatment plants, biosensors can report on the effect of operating conditions on HGT rates and the diversity of bacteria that participate in conjugation. **(C)** HGT biosensors can couple the production of a visual reporter to HGT. With this approach, the donor cells keep the reporter production off, while the receiver cells are unable to repress reporter production. Cells acquiring a conjugative plasmid (cPlasmid) through HGT produce an output that can be quantified using flow cytometry. **(D)** Biosensors can record a HGT event by writing new sequences into their DNA (red), which can be read out using qPCR or sequencing at later times provided that the DNA remains extractable.

Microorganisms adapt and evolve by transferring genetic materials through HGT (Andam et al., 2015). The three mechanisms of HGT include conjugation, transformation, and transduction. During conjugation, one microbe transfers DNA to another through direct contact (Figure 4A). Environmental conditions, like hydration and heterogeneity, can influence the frequency of direct microbe-microbe interactions. A wide range of genes are transferred through conjugation, including genes that underlie nitrogen fixation, photosynthesis, degradative catabolic processes, virulence, heavy metal and antibiotic resistance, and biofilm formation (Boucher et al., 2003; Aminov, 2011; Andam et al., 2015; Abe et al., 2020). This diversity of exchanged genes makes HGT studies critical to understanding elemental cycling in many environmental niches, including the cycling of nitrogen in soils (Bailly et al., 2007; Andam et al., 2015), carbon processing in oceans (Zimmerman et al., 2020), and the dissemination of antibiotic resistance in terrestrial and aquatic environments (Figure 4B), including wastewater (Karkman et al., 2017).

Most methods for studying conjugative HGT are disruptive to environmental samples, and this limits our ability to study HGT *in situ*. The most common method for measuring HGT involves the addition of a selective marker into the conjugative plasmid so that cells acquiring the plasmid (transconjugant cells) can be selectively cultured and quantified (van Elsas and Bailey, 2002). This method cannot provide dynamic information on transfer frequencies in environmental materials and cannot distinguish between the signal coming from newly formed transconjugants arising from HGT versus cells arising from vertical gene transfer that occurs when a plasmid-containing cell reproduces.

Some advances have been made in using fluorescent reporters to track HGT (Figure 4C). In one approach, a donor strain is programmed to repress a visual output until it is transferred to receiver cells, allowing the fraction of receiver cells acquiring the conjugative plasmid to be rapidly quantified using cell sorting (Pinilla-Redondo et al., 2018). Using this approach, the permissiveness of diverse recipient strains toward broad host plasmid conjugation has been characterized in soil (Klümper et al., 2014) and aquatic communities (Arias-Andres et al., 2018). This approach has also been used to understand how environmental parameters (e.g., nutrients, hydration, and stressors) impact the rate and range of conjugation (Musovic et al., 2014; Klümper et al., 2017).

Biosensor studies have revealed that some conjugation systems are differentially influenced by environmental conditions. Conjugation systems that rely upon either a flexible or rigid conjugative pili for mating show opposing trends in soils under different hydration conditions (Cheng et al., 2016; Tecon et al., 2018). This suggests that accurate models predicting HGT under different climate change scenarios will require advanced biosensor studies on how HGT is influenced by environmental conditions.

Despite advances in biosensors, our ability to precisely track HGT arising from conjugation, transduction, and naked DNA uptake (transformation) remains limited. To understand how to limit deleterious HGT, such as the spread of antibiotic resistance, it is critical that we develop strategies to monitor the flow of genetic information through microbial communities *in situ*, such as the rate of genetic retention in a population and the role of key microbial players that are hyper-conjugative. To understand how to promote beneficial HGT, such as the exchange of plasmids that nitrogen-fixing microbes require for symbiosis with plants, simple strategies are needed to monitor how the physicochemical properties of an environment affect HGT.

Measurements of HGT rates can be challenging in any environmental context because HGT often occurs at low frequencies, and rates can vary significantly under the nutrient-limiting conditions that are widespread in the environment (Lorenz and Wackernagel, 1991). Two recent studies illustrate the potential for synthetic biology to obtain new HGT information. One study showed that gas reporters can provide real time data on transconjugant formation without extracting cells from a soil (Cheng et al., 2016). The other study showed that genetic systems can be programmed to record rare HGT events using DNA-encoded memory (Figure 4D). An *E. coli* was engineered to quantify HGT frequencies by recording DNA fragments acquired from foreign DNA at a single nucleotide level (Munck et al., 2020). This approach and other types of advanced biosensors that use synthetic memory elements are expected to help study other biological processes, such as the uptake of naked DNA and transduction rates in environmental samples (Liang et al., 2019).

### Biosensors Can Study Spatial Heterogeneity *in situ*

Most microbes on Earth evolved to live in environments with high spatial heterogeneity (Whitman et al., 1998; Kallmeyer et al., 2012). Spatial gradients are exploited by microbes to control movement via chemotaxis (Stocker, 2012), coexist as a community (Tecon and Or, 2017), and perform metabolic reactions (Stubbendieck et al., 2016). A key aim for biogeochemists and microbial ecologists is to understand how heterogeneity controls microbial processes that underlie larger-scale elemental cycling. Currently, it remains challenging to address such questions because existing techniques cannot provide information at the micron-scale where microbes perceive their environment (Baveye et al., 2018). As an example, -omics approaches generate large amounts of data on the biomolecules that are synthesized by a microbial community. However, these approaches typically sample environmental materials at the mm and cm scales (Armitage and Jones, 2019) rather than the μm scale where microbes sense and respond to their environment. Because of their size, biosensors can report on spatial heterogeneity of biomolecules at the micron-scale.

**Figure 5:**
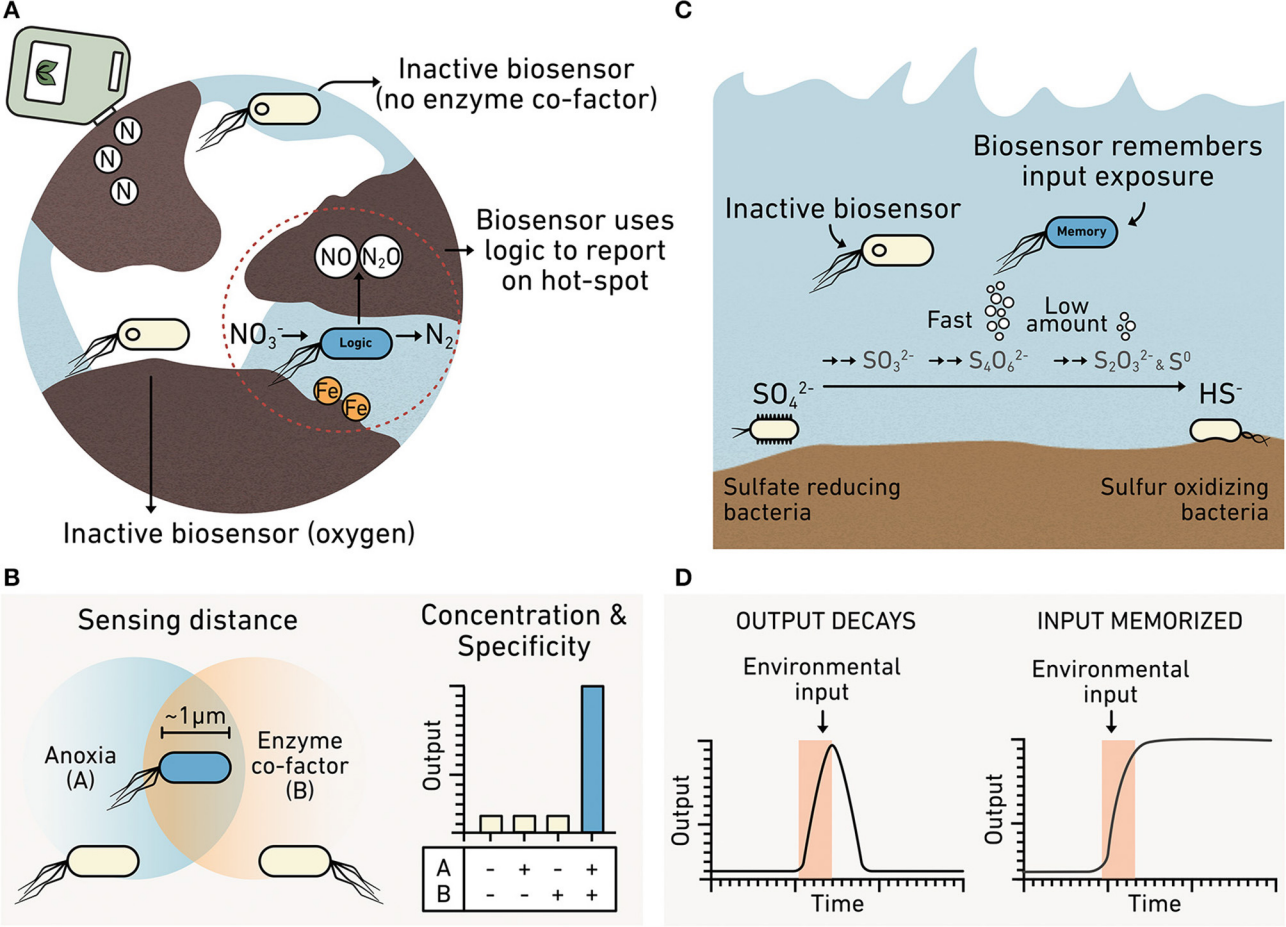
Biosensors can report on spatial and temporal heterogeneity. **(A)** A denitrifier programmed as a biosensor could be engineered to integrate information about multiple analytes such as the presence of iron required for nitrous oxide production and anoxic conditions. Cells unable to sense both iron AND anoxia remain inactive (*yellow*), while cells sensing both produce an output (*blue*). **(B)** Truth table showing how a biosensor with this AND gate logic only produces the reporter when input A (anoxic conditions) AND input B (iron) are colocalized at the micron scale. **(C)** Biosensors can monitor diverse intermediates in the sulfur cycle, including those that do not accumulate to high levels because they are rapidly consumed. **(D)** A comparison of biosensors that report in real-time or using memory. A real-time biosensor (*left*) only produces the reporter while the environmental input is present because the signal decays. This approach is hard to use when studying transient chemicals that are cryptic. A memory biosensor (*right*) converts information about transient signals into an output that is stable for long durations such that the biosensor memorizes the information.

Metabolic reactions that produce and consume greenhouse gases are influenced by environmental heterogeneity (Butterbach-Bahl et al., 2013). For these reactions to occur, electron donor and acceptor gradients must occur at relevant scales and be available for microbes to exploit (Groffman et al., 2009). Denitrification, the process that converts nitrate or nitrite to N_2_, generates N_2_O as an intermediate (Figure 5A), a greenhouse gas 300 times more potent than CO_2_ (Forster et al., 2007). Biological N_2_O production is dependent upon metal cofactors, such as iron and copper (Glass and Orphan, 2012). Additionally, even though denitrification occurs anaerobically, nitrogen cycling depends on processes that have different O_2_ requirements (e.g., nitrification), making the distribution of O_2_ a critical parameter for understanding how efficiently microbial communities carry out nitrogen cycle transformations. Studies of N_2_O release at the meter scale in soils have revealed large variations in N_2_O emissions that span more than an order of magnitude (Mathieu et al., 2006). This observation indicates that there is a need to better understand how the efficiency of denitrification is influenced by heterogeneity of metals and O_2_. Additionally, the impact of heterogeneity in marine systems on N_2_O production remains unclear. Marine O_2_ minimum zones result in N_2_O production hot spots (Kuypers et al., 2018). Synthetic microbes programmed to report on conditions that influence N_2_O production could help establish how spatial heterogeneity controls denitrification and subsequent N_2_O release (Venterea et al., 2012).

Field studies of CH_4_ emissions have also revealed high spatial variability (Sey et al., 2008), which needs to be better understood to manage this greenhouse gas. Methane is the final product of anaerobic degradation of organic matter, and methanogenesis is mostly driven by a select group of anaerobic archaea that live in diverse environments, such as saturated soils (e.g., rice paddy fields), animal rumens, the deep marine biosphere, and engineered systems (e.g., digesters and landfills). As a consequence, methanogenic archaeal physiological tolerance can span a wide range of environmental conditions, including diverse pH, temperature, and osmolarity ranges (Aronson et al., 2013). Their metabolic responses also depend on the availability of electron donor sources (e.g., hydrogen or small organic molecules) that can vary spatially in the environment (Enzmann et al., 2018). A better understanding of how spatial and temporal variations in environmental conditions affect CH_4_ cycling will enable improved predictions of fluxes of this greenhouse gas under different climate change scenarios (Bardgett et al., 2008).

New tools in synthetic biology could be useful in understanding key control points in microbial behaviors within natural chemical gradients (Figure 5B). It is unclear which of the many microbially-sensed chemical gradients are the final controls on the production of the greenhouse gases N_2_O and CH_4_. Sensor modules for diverse carbon and nitrogen cycle intermediates have been identified (Table 1) and could be incorporated into more sophisticated circuits. For example, synthetic microbial AND gates could be used to simultaneously integrate information on the presence of several environmental conditions occurring at the micron scale. Engineered denitrifiers and methanogens that couple the sensing of two or more environmental conditions in parallel to the generation of a single output can be used to establish how different chemical gradients act in parallel to regulate microbial behaviors through the creation of “hot spots” of metabolic activity (Chien et al., 2019). Microbial AND gates have been used to detect combinations of heavy metals, divalent cations, and carbon sources (Wang et al., 2013; Chien et al., 2019). Currently, AND gates are needed that report on conditions thought to control CH_4_ and N_2_O production, such as the expression of: (1) enzymes responsible for carbon and nitrogen processing, (2) proteins that mediate metal uptake and build the metal cofactors on those enzymes, and (3) bioavailability of metal cofactors (Glass and Orphan, 2012).

Some metal cofactors in carbon and nitrogen processing enzymes are O_2_ sensitive (Outten and Theil, 2009). Thus, biosensors with AND gate logic that report on the spatial overlap of O_2_ and metals would be useful for understanding how nutrient availability, O_2_ stress, pH variability, and metal limitations influence greenhouse gas production. The information provided by biosensors could also provide insight into the ways that the chemical composition of a microbe's niche relates to the average composition of an environmental sample. When implemented in denitrifiers and methanogens, such biosensors would allow measurements that examine how chemical gradients affect growth, resource acquisition, stress responses, and carbon and nitrogen transformations. Such information is needed to improve models that predict future scenarios of greenhouse emissions under climate change (Gattuso et al., 2018; Cavicchioli et al., 2019; Malik et al., 2020).

While biosensors hold great potential for studying heterogeneity *in situ*, there are still significant challenges with their use in natural matrices. First, there remain technical and ethical hurdles to introducing synthetic microbes into the environment. Cell-free reactions sidestep these challenges by using non-living biomolecular components. These reactions can be freeze-dried or fixed to paper, resulting in biosensors that can sense an environmental input and produce a pigment or fluorescent output, much like a pH strip (Pardee et al., 2014). Cell-free biosensors are easily used in the field (Jung et al., 2020) and could facilitate *in situ* measurements of cm-, m-, or km-scale gradients that underlie heterogeneity in N_2_O and CH_4_ production (Mathieu et al., 2006; Sey et al., 2008).

### Biosensors Can Record Hard-to-Observe Reactions

Because biosensors detect at the temporal scales of microbial behaviors, they hold the potential to monitor fast chemical transformations in the environment that are hard to observe, often described as cryptic (Figure 5C). Currently, there is a need to understand cryptic reactions related to iron and sulfur cycling, because they play key roles in the decomposition of organic matter and in the production of greenhouse gases. For example, the sulfur cycle is thought to control ~29% of carbon mineralization in marine sediments (Wasmund et al., 2017). The interconversion of sulfur intermediates happens rapidly enough that conventional methods only report on sulfate and hydrogen sulfide concentrations; they cannot report on the transient chemical intermediates underlying the marine sedimentary S cycle (Orphan, 2009; Jørgensen et al., 2019). Existing techniques can provide insight into the metabolic activities of uncultured microbes *in situ* by coupling secondary ion mass spectroscopy to fluorescence *in situ* hybridization (Morris et al., 2013). However, acquiring time series data is sample and resource intensive when performing studies across the hour, day, week, month, and seasonal time scales.

Like the sulfur cycle, the iron cycle is hard to track because it is controlled by a variety of rapid biotic and abiotic reactions, including photochemical reduction, aerobic oxidation, and biotic transformations of iron mediated by ferric-reducing and ferrous-oxidizing bacteria (Peng et al., 2019). The rapid cycling between ferric and ferrous ions is also hard to monitor *in situ* due to low concentration of these ions (Kappler and Bryce, 2017). Synthetic biology is poised to enable new dynamic studies of cryptic processes across many time scales. Microbes can be engineered to respond to a cryptic metabolite through either post-translational or transcriptional control on the millisecond to hour time scales (Olson and Tabor, 2012).

The simplest way to leverage synthetic biology to study cryptic cycling is to introduce “spy” microbes into communities for short periods of time, coding them to report on a hard-to-observe metabolite by producing an easily measurable output in real time. In a more advanced approach, microbes that participate in cryptic processes could be engineered as biosensors directly, although this is currently challenging because many have yet to be cultured. One way to overcome this challenge is to program unculturable microbes within the context of their natural communities (Ronda et al., 2019). This could be achieved by programming conjugative plasmids with sensors for detecting target metabolites in, for example, the sulfur cycle and using HGT within an environmental sample to program the microbes of interest. In the case of the sulfur cycle, sensors for thiosulfate, tetrathionate, and hydrogen sulfide have been described (Daeffler et al., 2017; Liu et al., 2019). Sensors that report on extracellular ferrous and ferric ions have also been used measure iron fluctuations in spatially and temporally heterogeneous environments such as the rhizosphere and ocean waters (Joyner and Lindow, 2000; Lam et al., 2006). In cases where natural components are used to build sensors, many may require tuning to achieve the desired sensitivity range. The chemical concentrations required to trigger responses can now be tuned in some sensing systems.

It is also now possible to record information about the transient production of a cryptic chemical by one microbe prior to the consumption of that metabolite by a second microbe. This recording (Figure 5D) can be achieved by programming microbes to modify their DNA after exposure to a chemical as an output (Munck et al., 2020). This DNA-coded information can then be read out after incubations of varying duration to reveal what metabolites an individual microbe encountered and the order of exposure to those metabolites. With this approach, one advantage is that biosensors record information that can be retrieved even after cell death, provided that the modified DNA can be extracted from an environmental sample (Dai et al., 2017).

## B. State of the Field

Simple biosensors have been used to address environmental questions for over 30 years, and a number of excellent articles review historic work (Gage et al., 2008; van der Meer and Belkin, 2010). As outlined in section A, diverse environmental and Earth science questions could be addressed using emerging synthetic biology capabilities. In this section, we discuss recent innovations including the expansion of sensing capabilities, diversification of outputs, creation of more sophisticated genetic circuits, and the development of approaches for tuning circuit components for environmental applications.

### Inputs: Sensing Capabilities Can Monitor Diverse Conditions

Sensing modules have been engineered that respond to a broad range of environmentally-relevant organic and inorganic chemicals as well as community behaviors like HGT and cell-cell interactions. While many early biosensors were designed to sense toxic chemicals (King et al., 1990), modules have also been developed to sense chemicals integral to biogeochemical cycles (Tables 1–3). With these new sensing capabilities, biosensors can now be applied to answer fundamental questions about how microbes perceive fluctuations in both chemical and physical environmental parameters.

All biosensors have two essential functions: (1) to respond to an environmental condition, and (2) to translate that response into a measurable output (Daunert et al., 2000). While sensing modules can use a wide range of biological architectures to convert environmental information into biochemical information, they typically function as either intracellular or extracellular switches.

#### Intracellular Sensors

**Figure 6:**
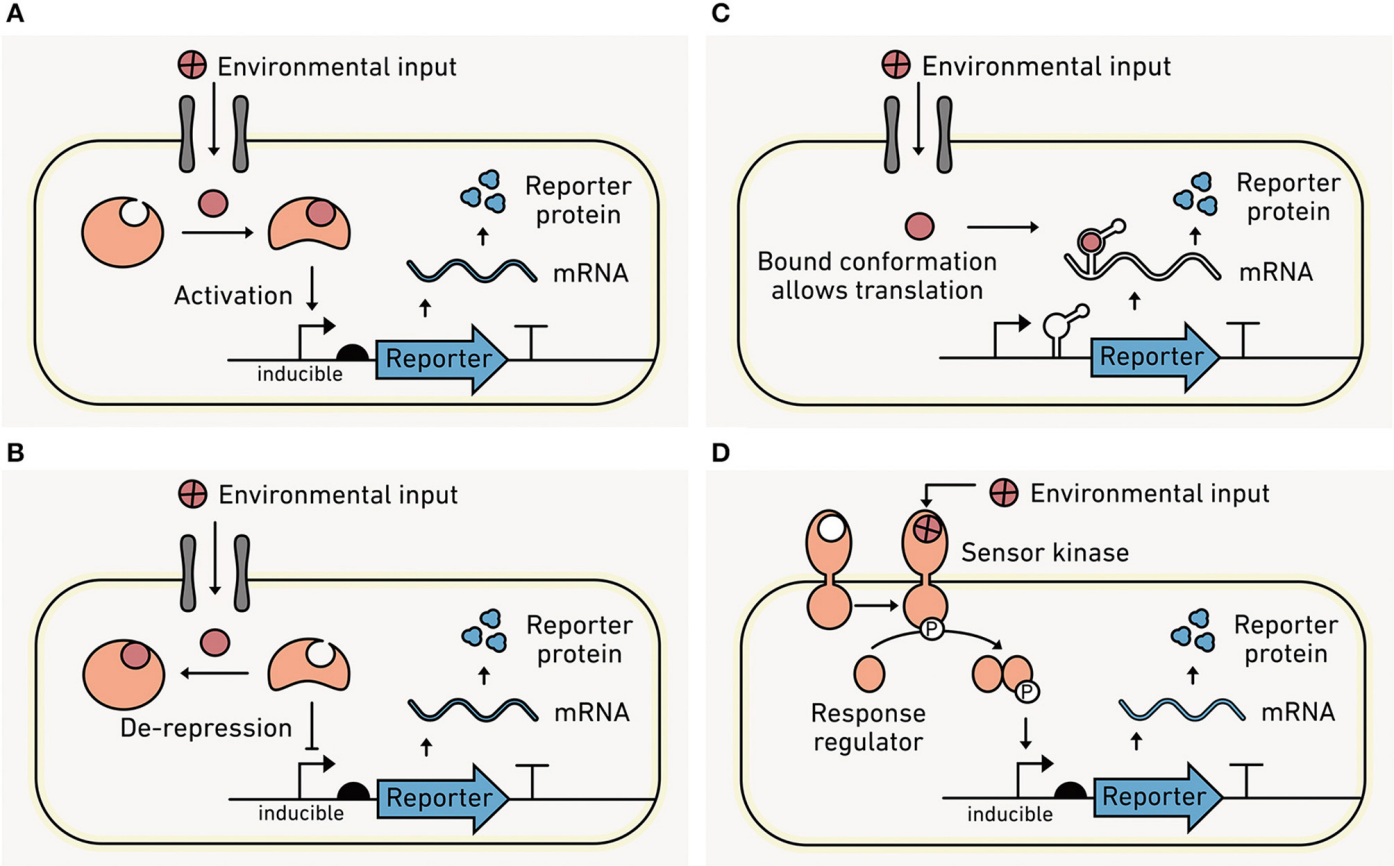
Sensing intracellular and extracellular conditions. **(A)** With intracellular sensors, an environmental analyte (red) must cross the cell membrane into the cell via diffusion or through a transporter to be detected. Analyte binding to a sensor protein in the cytosol increases protein-DNA affinity and leads to the recruitment of RNA polymerase and output transcription. **(B)** Intracellular analyte binding can also enhance transcription by causing dissociation of protein from the DNA that is blocking output transcription. **(C)** Intracellular analyte binding to a riboswitch can trigger a conformational change near the ribosomal binding site (RBS) that permits ribosome binding and translation of the output module. **(D)** Extracellular sensors bind to the analyte using surface-exposed proteins such as the sensor kinase from a TCS. Following analyte binding, the kinase phosphorylates a response regulator within the cell, which binds to DNA and alters output transcription.

Protein or RNA switches within cells sense an environmental condition and couple that sensing to a change in transcription that regulates reporter output production. Protein-based sensors often use transcription factors, which evolved to sense a wide range of analytes (Tables 1–3). With these sensors, analyte binding induces a conformational shift in the transcription factor (Figures 6A,B) that changes its interaction with DNA. DNA binding subsequently alters gene expression. Transcription factors can also be evolved or engineered to sense new environmental inputs (Libis et al., 2016). In addition to transcription factors, CRISPR-based sensors can be used to detect specific DNA or RNA sequences (Gootenberg et al., 2017). Such approaches have recently been developed as tools for point-of-care diagnostic for malaria species (Lee et al., 2020).

RNA molecules can also be engineered as intracellular sensors (Figure 6C). Like protein switches, RNA switches (called riboswitches) use analyte binding to drive conformational changes that affect gene expression (Mironov et al., 2002; Nahvi et al., 2002). Natural riboswitches have been discovered that interact with organic metabolites and inorganic ions (Serganov and Nudler, 2013), and synthetic riboswitches have diversified to sense different organic chemicals (Xiu et al., 2017) and temperature (Sen et al., 2017). It is important to emphasize that these switches uniformly sense environmental conditions *within the cell*, information that is useful for connecting environmental conditions to cellular responses. However, they are sensing information distinct from extracellular environmental conditions.

#### Extracellular Sensors

Local conditions outside of the cell can be converted into biochemical information within the cell through surface-exposed biomolecules. Two component systems (TCSs) represent a major class of extracellular sensors (Figure 6D). TCSs consist of a membrane-bound kinase that senses an environmental condition and transmits this information to an intracellular response regulator protein which modulates gene expression. With gram-negative bacteria, the sensor kinase is within the inner membrane, so analytes must pass through a pore protein in the outer membrane prior to binding, which has a size limit of ~500 amu. Gram-positive bacteria do not have the same limitation because the sensor kinase is directly exposed to the cell surface.

TCSs are widely distributed across prokaryotes and have evolved to sense a diverse range of environmental conditions (Capra and Laub, 2012). Consequently, TCSs have been extensively repurposed as biosensors in synthetic biology (Tables 1–3). Additionally, protein engineering has yielded simple strategies to couple the sensing by diverse TCS systems to a standardized output, facilitating the process of TCS discovery and accelerating the process of repurposing TCS in biosensors (Schmidl et al., 2019).

### Outputs: Environmentally-Compatible Reporters Are Emerging

**Figure 7:**
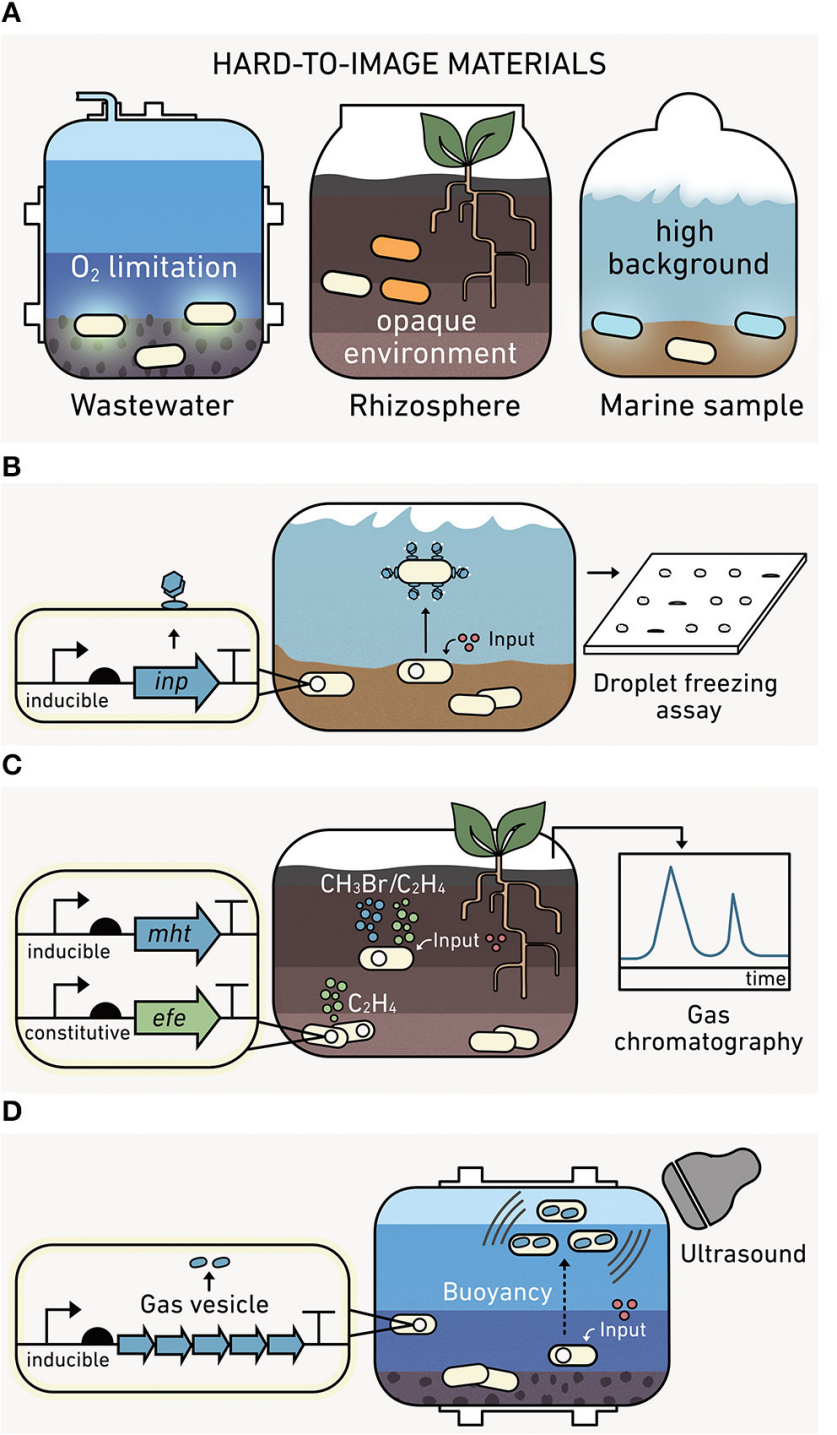
Output modules enable reporting from environmental materials. **(A)** Visual outputs are hard to use in environmental materials. (*left*) Fluorescent proteins require oxygen to mature, which limits their use in anoxic conditions. (*middle*) Pigment-producing proteins are hard to image in soils and sediments. (*right*) Bioluminescence can have a high background in marine samples. **(B)** The ice nucleation protein module (*inp*) is compatible with matrix experiments. Following biosensor production, INP levels can be measured from water extracted from a sample. **(C)** Indicator gas outputs can be monitored in the headspace of environmental materials using gas chromatography without sample disruption. The methyl halide transferase (*mht*) and ethylene forming enzyme (*efe*) modules synthesize methyl halides and ethylene, respectively. One gas (green) reports on cell growth, while the second gas (blue) provides information on the analyte detected. The ratio of these gases provides a robust output because it represents the average analyte sensed per cell. **(D)** Gas vesicle outputs are encoded by an acoustic operon, which yield a unique ultrasound signal upon expression.

Ideally, outputs should allow non-disruptive, real-time reporting in environmental materials like soils, marine sediments, and wastewater. While visual reporters such as fluorescent proteins (Shaner et al., 2005), luminescent proteins (Saito and Nagai, 2015), or pigment-producing enzymes (Watstein et al., 2015; Verosloff et al., 2019) provide spatial information when cells are visualized through microscopy (DeAngelis et al., 2005; Pini et al., 2017), they are hard to use in opaque materials like soils and sediments (Figure 7A). Even in liquid marine and lacustrine systems, high autofluorescence from native organisms, dissolved organic carbon, and other chemicals can obscure outputs from visual reporters (Carstea et al., 2020). The heterogeneity of O_2_ in many environments also presents challenges because common green fluorescent protein (GFP) reporters require O_2_ to fluoresce (Heim et al., 1994). To address these challenges, environmental biosensors should have low background signals, signal intensities that are easy to calibrate without sample disruption, and activities across gradients of O_2_ levels.

#### Ice Nucleation Protein (INP)

One of the earliest alternatives to visual reporters for environmental studies was INP (Figure 7B), which catalyzes ice formation from supercooled water (Lindgren et al., 1989). INP outputs from biosensors incubated in soils can be retrieved through washing soil and assessing INP in the aqueous fraction using a droplet freezing assay (Loper and Lindow, 1994; Jaeger et al., 1999; DeAngelis et al., 2005). INP can be used to report under both aerobic and anaerobic sample conditions (DeAngelis et al., 2005). However, INP only provides information through a single channel, making it hard to differentiate if a signal arises from many cells expressing a small amount of INP versus a few cells making large amounts of INP.

#### Gas Reporters

Indicator gas outputs do not require destruction of an environmental matrix to observe the output signal, since they can be measured in the headspace of samples using gas chromatography mass spectrometry (Figure 7C). To date, microbes have been programmed to synthesize a wide array of volatile gases, including C_2_H_4_O (Bongers et al., 2005; Zhu et al., 2011), CH_3_Cl, CH_3_Br, and CH_3_I (together referred to as CH_3_X) (Cheng et al., 2016), C_2_H_4_ (Cheng et al., 2018), and H_2_S (Bang et al., 2000). However, only CH_3_X and C_2_H_4_ have been used for environmental biosensing. CH_3_X has been used to report on conjugation in soil (Cheng et al., 2016). Additionally, CH_3_X and C_2_H_4_ have been used in parallel to report on the activity of a sensing module (a conditional CH_3_X output) and the number of microbial biosensors (a constitutive C_2_H_4_ output) performing the sensing (Cheng et al., 2018). By calculating the ratio of these gases, challenges calibrating what fraction of the CH_3_X arises due to biosensor growth are avoided. Among the various indicator gas outputs, three (C_2_H_4_O, CH_3_X, and H_2_S) function in both aerobic and anaerobic conditions (Bang et al., 2000; Cheng et al., 2016; Balagurunathan et al., 2018), while C_2_H_4_ requires O_2_ for synthesis (Cheng et al., 2018).

#### Gas Vesicles

Cyanobacteria regulate buoyancy by expressing protein-structures that fill with gas (Pfeifer, 2012). These structures were recently used to create a new type of output called gas vesicles (Figure 7D). These vesicles have been expressed in non-native microbes and used as contrast agents for hard-to-image vertebrate models (Shapiro et al., 2014). When microbes expressing these vesicles are placed in communities within a hard-to-image animal gut, they can be visualized non-invasively using ultrasound or magnetic resonance imaging (Lu et al., 2018; Farhadi et al., 2019). While these outputs have not yet been used in environmental biosensors, they are expected to be useful as a contrast agent in wastewater when imaged using ultrasound.

### Processing Modules: New Tools Can Record Complex Information

Synthetic biology is rapidly moving beyond the one-input, one-output biosensor paradigm. The growing catalog of parts available for engineering biosensors now enables complex logical operations and the ability for cells to record their own experiences. This section introduces logic gates that use multiple inputs and memory systems that expand the complexity and capabilities of environmental biosensing. These capabilities open up new opportunities to study spatial and temporal heterogeneity (see section B).

#### Logic Operations

**Figure 8:**
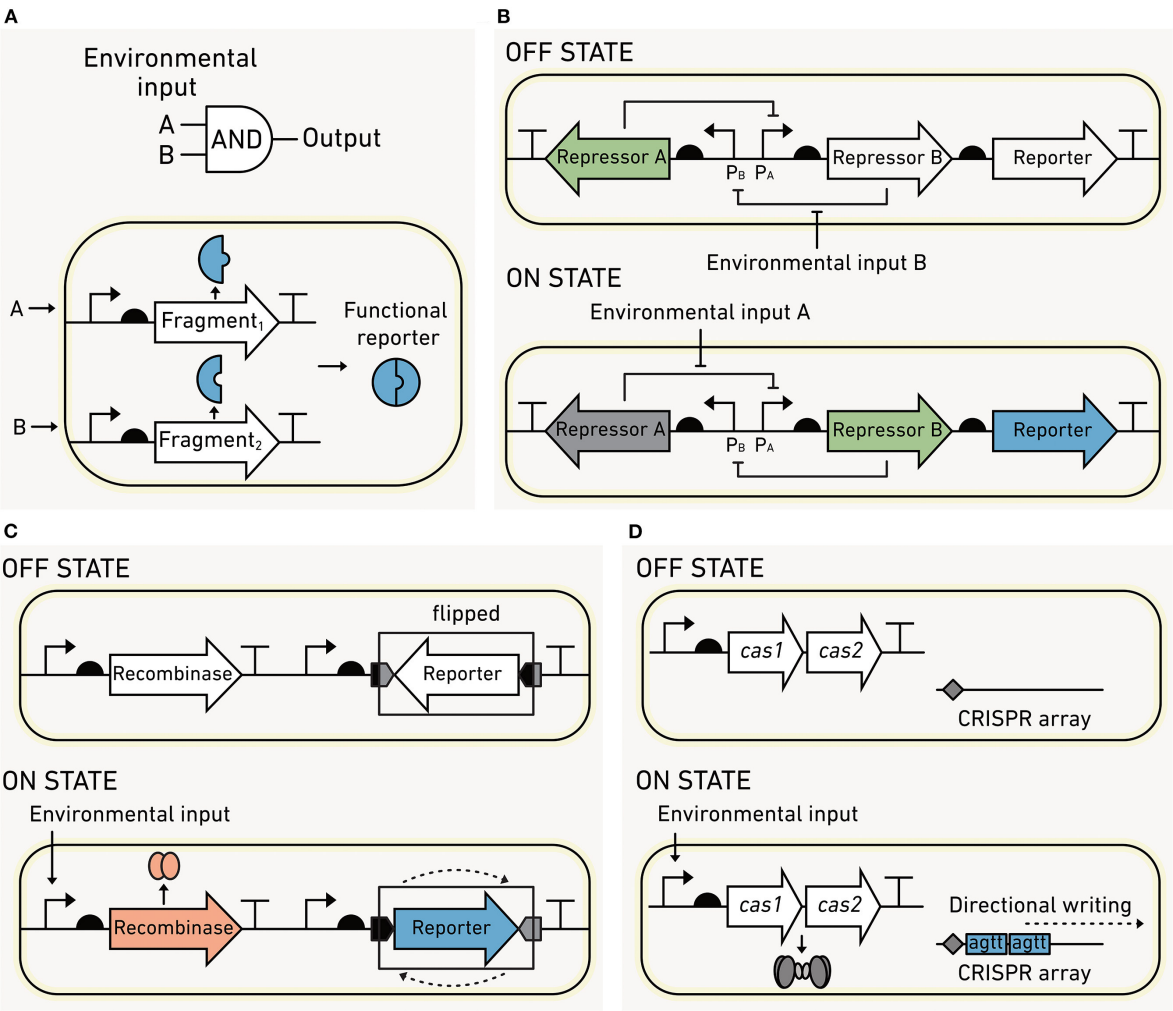
Processing modules can increase biosensor sophistication. **(A)** Two-input AND gates can be created by using modules that encode protein in fragments. The output only occurs when the first AND second fragment are produced. **(B)** Flexible DNA memory using a toggle switch. In the OFF state, transcriptional repressor A blocks the production of the repressor B and the reporter output. Upon sensing environmental input A, the circuits flip to an ON state where repressor A is momentarily dissociated from promoter P_A_, allowing the production of repressor B and the output. Repressor B blocks production of repressor A, stabilizing this ON state. The switch can be flipped back to the OFF state upon sensing of environmental input B. **(C)** Fixed DNA memory built using recombinases. In the OFF state, no recombinase is made, and the reporter DNA sequence is antiparallel to the regulatory elements required for expression. In the ON state, an environmental input induces recombinase production, which binds a pair of DNA sequences flanking the reporter and inverts the DNA such that the reporter is parallel to the regulatory elements. This flipping of the DNA leads to output production. **(D)** Fixed DNA memory that uses CRISPR. Input sensing is coupled to the expression of Cas1/2, which incorporates short DNA spacers into a CRISPR array at a rate that is proportional to the input exposure.

In synthetic biology, multiple inputs can be combined into a single output following logical operations (AND, OR, NOT). For example, a two-input AND gate (Figure 8A) only produces the output if the first AND the second inputs occur in a cell's environment simultaneously (Brophy and Voigt, 2014). Numerous design strategies can be used to combine the presence (or absence) of multiple inputs into a single output (Shis and Bennett, 2013; Shis et al., 2014; Kim et al., 2019; Fulk et al., 2020). Processing modules can be constructed by adding a cascade of transcription factors between the input and output modules whose effects combine to produce the desired logic (Stanton et al., 2014). While diverse logic gates can be reliably built, only a small number have been applied in environmental contexts. One study showed that bacterial colonization of the mouse gut depends upon O_2_, pH, and lactic acid concentrations (Chien et al., 2019). Logic gates could similarly be used in other environmental contexts, for example to report on input combinations leading to greenhouse gas production.

#### Biological Memory

Cells can be programmed to record their past experiences by converting sensing information into heritable changes. Biological memory is either “permanent” (written into the microbe's DNA in a way that cannot be reversed) or “rewritable” (recorded in a way that enables it to be reset). Memory is appealing for environmental biosensing because it can be read out from both living and dead cells using DNA sequencing or quantitative polymerase chain reaction (qPCR) (Sheth and Wang, 2018). Below we review the first synthetic memory described (the toggle switch) and three new DNA-based recording systems that have promise for environmental studies.

#### Rewriteable Memory

With this memory, a pair of output states is used to indicate the most recent experience of a cell. In a classical example called the “toggle switch” (Figure 8B), the interconversion between the OFF (no output) and the ON (output) states is triggered by a small molecule in the environment. Each state is determined by the relative abundance of two transcriptional regulators. In the OFF state, the first protein regulator is made, and it inhibits the synthesis of the second protein regulator. The switch is flipped when the first regulator binds a chemical and can no longer inhibit the production of the second regulator. At this point, the second regulator accumulates in the cell and inhibits the synthesis of the first regulator. The memory circuit can also be switched from ON back to OFF by the addition of a second molecule through a similar mechanism (Gardner et al., 2000). Since both transcriptional regulators are partitioned into daughter cells upon cell division, the stable state is inherited across generations. Biosensors containing toggle switches have been deployed in the mouse gut to record molecular signatures of inflammation and antibiotic exposure (Kotula et al., 2014; Riglar et al., 2017). This memory has been shown to stably remain OFF for up to 6 months in the mouse gut, indicating that toggle switches may be feasible for recording experiences on this timescale (Riglar et al., 2017). However, protein-based memory states are lost upon microbial death or during dormancy, limiting their use to living cells.

Rewritable memory can also be coded as sequence changes in DNA. DNA-based memory circuits can report via production of a detectable signal, like toggle switches, or the memory output can be read directly from the DNA sequence. The DNA memory can be programmed using recombinases, enzymes that bind specific DNA sequences and mediate recombination at those sites (Ham et al., 2006). In most recombinase-based memory circuits (Figure 8C), the environmental signal induces recombinase expression. The recombinase inverts the segment of DNA that is within the DNA sequences that it binds. The flipped DNA output can be monitored by DNA sequencing, qPCR, or by incorporating a reporter gene that is turned on following the sequence change (Yang et al., 2014). It is possible for DNA recombinases to record both rewritable (Bonnet et al., 2012) and permanent (Yang et al., 2014) memory. In contrast to toggle switch memory, recombinase sequence modifications are inherited across generations and can be read out following cell death (Munck et al., 2020).

#### Permanent Memory

DNA recombinases that catalyze irreversible sequence inversions are a common mechanism of permanent memory (Yang et al., 2014), which cannot be erased. To date, more than 10 different recombinases have been identified, enabling the simultaneous recording of multiple inputs and the order of exposure to those inputs (Friedland et al., 2009; Roquet et al., 2016). Recombinase-based memory can also be used with logic gates to record combinations of environmental inputs (Siuti et al., 2013; Yang et al., 2014). This type of memory has been used to record the exposure of biosensors to sugar in the mouse gut. In this complex environment, the memory circuit was able to be accurately read for up to 4 days (Mimee et al., 2015). Recombinase memory is advantageous due to its simplicity and low cellular burden. However, it is susceptible to false positives in the absence of environmental inputs (Mimee et al., 2015).

In contrast to recombinase memory, which only modifies DNA at specific sequences, flexible DNA sequence recording enables precise modifications at diverse chromosomal locations. This approach is also capable of writing more information to DNA compared with recombinases. One flexible sequence recording strategy uses CRISPR-associated proteins to incorporate short DNA fragments (spacer sequences) into plasmids (Figure 8D), which functions as “recording tape,” growing in size at a rate proportional to the input signal. Different spacer sequences can be used in parallel to report on multiple environmental inputs (Schmidt et al., 2018). DNA sequencing can be used to read out the numbers and types of inserted spacers, and this information can be used to reconstruct the temporal order of sensed inputs (Sheth et al., 2017). This system has recorded the approximate time, duration, and order of cell exposure to copper, trehalose, and fucose over 6 days as well as HGT in fecal and gut samples (Sheth et al., 2017; Munck et al., 2020). While this memory has recorded information for 10 days in a lab setting (Zou and Ye, 2020), there is a need to determine whether this type of recording can provide information over a similar duration in complex environmental samples. Additionally, limitations of the maximum amount of sensing information that can be recorded and reliably differentiated by sequencing need to be established.

### Tuning: Refining Circuits for Robust Performance

To be useful in environmental studies, biosensors must sense and report robustly across complex, dynamic conditions that are less ideal for fast growth as compared to sterile, well-mixed laboratory cultures. Performing well in environmental conditions requires that biosensor inputs are sensitive to the relevant range of environmental conditions and specific enough to only sense the desired condition. Furthermore, biosensors outputs need to report with a high enough signal-to-background (or *gain*) to be reliably detected. To achieve these performance characteristics, biosensors may require multiple tuning steps and should be rigorously tested in the intended environmental context, i.e., a soil matrix or water sample, for false positive or false negative sensing.

#### Tuning Input Specificity

**Figure 9:**
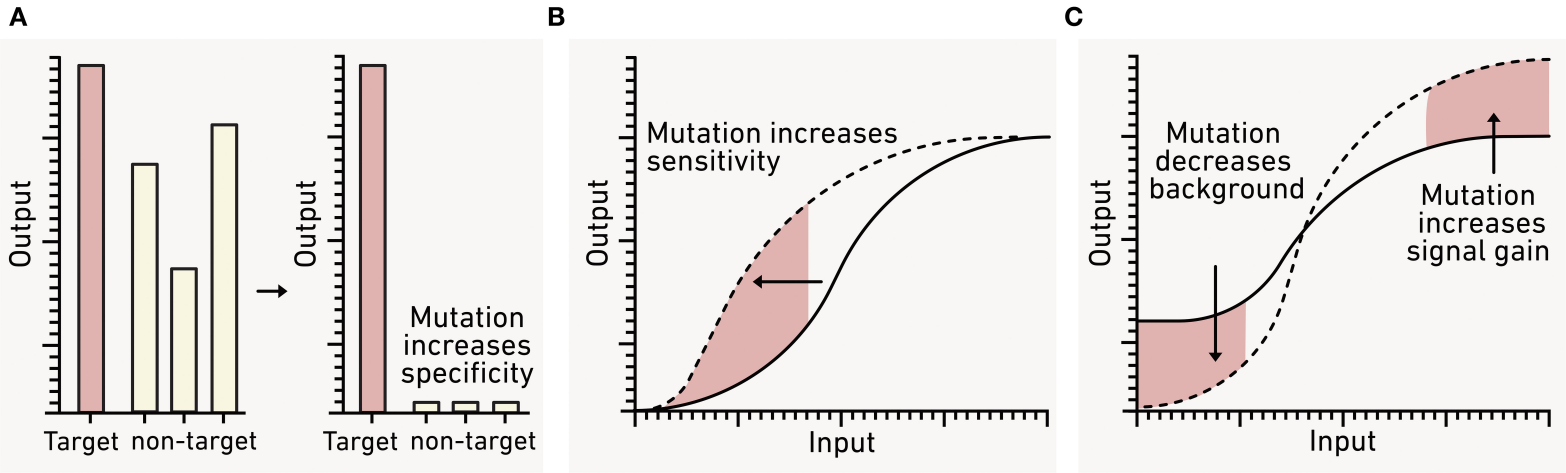
Using genetic engineering to tune biosensor performance. **(A)** Natural sensor modules often report on both target (*red*) and non-target (*gray*) analytes. To avoid false positives from the latter, sensors can be engineered to respond to a single input by incorporating mutations. **(B)** The sensitivity of a biosensor for a given analyte can also be adjusted by mutating the sensor module to adjust the transfer function (*dashed line*). **(C)** To improve the dynamic range, the biosensor background in the absence of analyte (OFF state) and the maximum signal (ON state) can also be tuned using mutation.

In heterogeneous environments, it is important for biosensors to specifically respond to the signal of interest (De Paepe et al., 2017). Many natural input modules are activated by chemicals with related structures (Figure 9A), which can inhibit desired inputs and create false negatives or produce unintended outputs that are false positives (Meyer et al., 2019). Environmental matrices often contain diverse chemicals with similar structures that have the potential to non-specifically activate biosensors (Dewhurst et al., 2002; Hawkes et al., 2016). For this reason, it is essential to carefully tune and test biosensor input specificity. Input modules that have suboptimal specificity can be improved through random mutagenesis followed by screening the resulting library for module variants with desired properties. This approach has yielded sensors that are specific to individual AHL signals, isomer-specific sugars, and organic acids (Collins et al., 2005, 2006; Tang et al., 2008; Tashiro et al., 2016; Snoek et al., 2020). Sensor modules can also be improved by recombining natural sensors to create chimeric variants. This approach has yielded sensors for plant-microbe signals and nitrogen cycle intermediates (De Paepe et al., 2019; Schmidl et al., 2019).

Environmental matrices in soils, sediments, or wastewater contain chemicals that have the potential to non-specifically activate biosensors. While we can begin to assess specificity by screening biosensors against a library of pure compounds, it is important to test new biosensors in environmentally-relevant chemical backgrounds containing microbial communities (Bereza-Malcolm et al., 2015). This can be achieved with control reactions that evaluate environmental backgrounds such as the ensemble of chemicals found in natural dissolved organic matter. Such control experiments are needed to establish and mitigate the impact of environmental effects on biosensor performance (McNerney et al., 2019).

#### Tuning Input Sensitivity

To be useful, biosensors must report on environmentally-relevant concentrations of the targeted analytes. To achieve this, input modules must be tuned so that they respond to the desired analyte concentrations (Figure 9B). In the environment, the bioavailable concentration of a chemical of interest can be influenced by sorptive processes, abiotic chemical reactions, and biotic transformations (McNerney et al., 2019). Additionally, output modules must be tuned so that they present a low background and a strong signal increase following input exposure (Figure 9C). This tuning is critical to ensure that the biosensor output is detectable over any environmental background, and that the difference in output production between OFF and ON states remains distinct over the relevant environmental time scale. These two goals are not mutually exclusive and are often addressed using similar strategies.

The simplest tuning method changes the number of circuit components synthesized in the cell (Brophy and Voigt, 2014). Frequently this tuning is achieved by increasing or decreasing the amount of sensor proteins synthesized. Depending on the specific needs, this tuning can be achieved by modifying sensor gene transcription, sensor protein translation, or sensor protein degradation (Salis et al., 2009; Huang et al., 2012; Brophy and Voigt, 2014; Cameron and Collins, 2014). When these efforts do not achieve the required performance, additional components like amplifier circuits can help improve the output-to-input ratio (Wan et al., 2019). Sensitivity can be improved by modifying the affinity of the sensor biomolecule for its signal or by altering the efficiency at which the sensor converts the input to into an output. This approach requires engineering the sensing molecule through rational design or directed evolution, similar to the approaches used to tune sensor specificity (Lönneborg et al., 2012; Landry et al., 2018; Meyer et al., 2019; Snoek et al., 2020).

## C. *In vitro* and *in vivo* Biosensors

The earliest biosensors were built by programming easy-to-manipulate and fast-growing bacteria like *E. coli* (Adams, 2016). While these sensors have been the mainstay of the biosensor field (Tables 1–3), there is a growing effort to diversify the types of organisms that can be reliably programmed using synthetic biology. Biosensors created from environmental microbes will be useful for reporting on new types of information within more realistic settings, for example, by enabling biosensors to report on their own experiences *in situ* within environmental matrices. Additionally, innovations in cell-free biosensors have recently enabled fast, easily deployable sensors that can monitor macroscale spatial and temporal heterogeneity at field sites, analogous to point-of-care diagnostics used in biomedical applications (Jung et al., 2020; Thavarajah et al., 2020).

### Engineering Relevant Cellular Chassis

**Figure 10:**
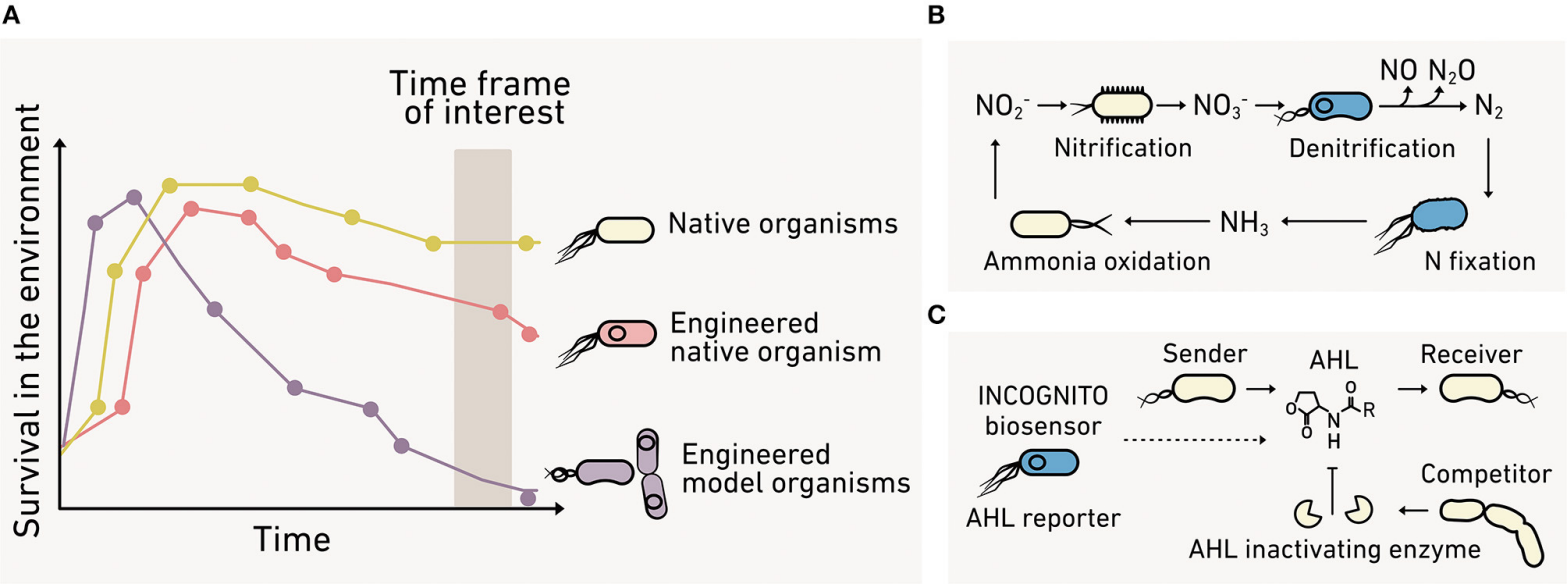
The benefits of engineering environmental microbes. **(A)** Biosensors made using model organisms (*purple*) can present poor fitness under environmental conditions compared with native organisms (*yellow*). Advances in synthetic biology make possible the programming of native organisms (*red*) to overcome this limitation. **(B)** Environmental microbes can be programmed to directly report their behaviors like contributions to the nitrogen cycle. **(C)** Alternatively, incognito biosensors can be created that spy on a community without interfering with the process of interest.

To study ecological processes, biosensors need to survive and perform as intended under realistic conditions (Figure 10A), which include large dynamic changes in temperature, hydration, pH, and substrate levels. Some applications would benefit from programming organisms that participate in a specific environmental process. A “super host” microbe that engages in many HGT events could be useful to study antibiotic resistance transmission through an environmental microbiome. Alternatively, a host that performs specific steps in a biogeochemical cycle could be applied toward understanding environmental controls on flux through carbon or nitrogen cycling pathways (Figure 10B). In other contexts, the biosensor may need to be “incognito” to achieve the desired sensing without interfering with the community being studied (Figure 10C). As an example, it would be useful to build biosensors using cells that do not produce or degrade cell-cell signaling molecules in cases where the goal is to monitor this communication in marine biofilm-associated microbial communities (Hmelo, 2017).

Over the past decade, synthetic biology has developed strategies to expand the number of microbes that can be programmed and biological parts that function reliably across diverse cellular chassis (Chi et al., 2019; Kim et al., 2020). Below we outline new methods available to program non-model organisms.

#### Incorporation of DNA Into Environmental Microbes

The introduction of foreign DNA into undomesticated microbes is challenging because DNA transformation methods require that individual strains be cultured, which is only possible for a small fraction of microorganisms (Steen et al., 2019). New approaches leverage conjugation to deliver genes directly into a microbial community without the need for strain isolation (Whitfill and Oh, 2019). Transconjugants arising from this approach are more likely to survive and thus are poised to perform well as biosensors. Following conjugation, synthetic DNA can be integrated into the chromosome or encoded on plasmids that propagate in the host of interest. *In situ* conjugation has been achieved in soil isolates using the gram-positive *Bacillus subtilis* as a donor of the mobile element ICE, which is able to integrate large fragments of DNA into gram-positive bacterial chromosomes (Brophy et al., 2018). A hyper-conjugative *E. coli* has also been used to conjugate into gram-positive and gram-negative bacteria within the mammalian gut microbiome (Ronda et al., 2019). The conjugative plasmid in this strain contained the IncPαRP4 replication and transfer system, which is expected to be useful in diverse environmental communities beyond the gut. New technologies also enable targeting of specific members of a microbial community for genome editing (Rubin et al., 2020).

#### Expression of Synthetic Circuits in Environmental Microbes

For synthetic circuits to function in environmental microbes, their modules must be synthesized using regulatory elements for transcription (promoters) and translation (RBSs) that function in those contexts. Regulatory elements from laboratory strains may not yield the desired activities in non-model organisms because the biomolecules that control transcription (RNA polymerases) and translation (ribosomes) vary across organisms. There are several approaches to build functional genetic circuits in non-model organisms, including (1) the use of native promoters and RBSs that have been characterized in the strain of interest, (2) the implementation of non-native regulatory elements that function independently from native systems, and (3) the use of regulatory elements that present similar functions across a wide range of organisms.

**Figure 11:**
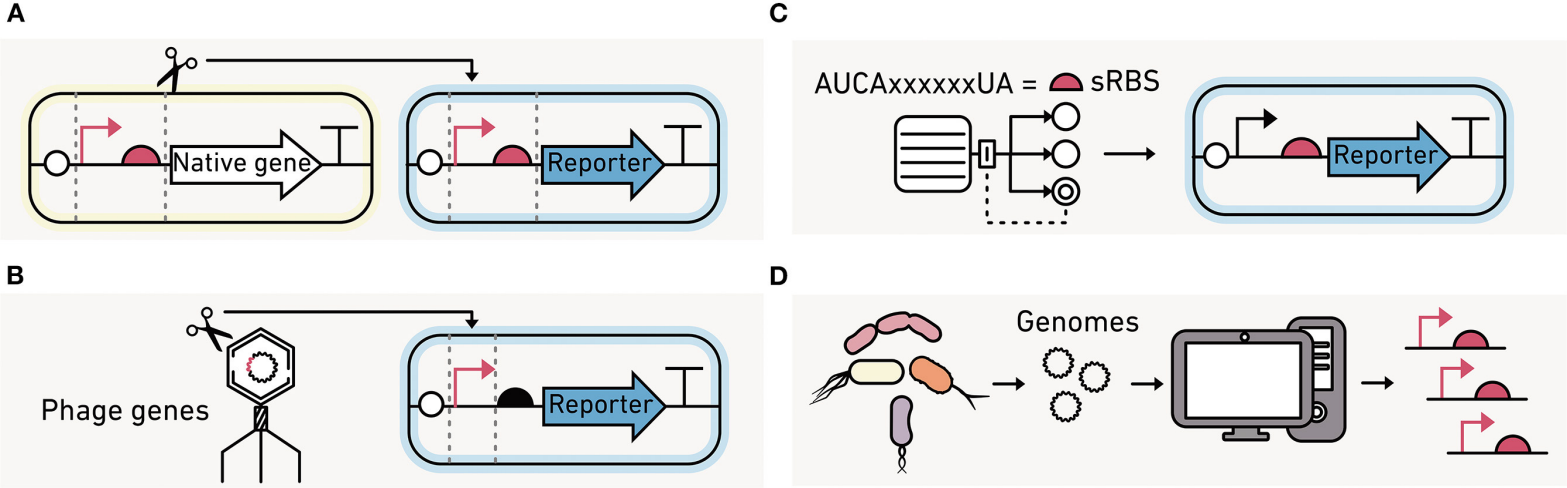
Strategies for identifying regulatory elements to program environmental microbes. **(A)** Native promoters and RBS from the organisms being engineered can be used to build the synthetic circuits if their activities are known. **(B)** Promoter and polymerase pairs that are functionally orthogonal from those in the native organisms can be used to control transcription, such as those encoded by phage. **(C)** Computational models can design synthetic RBS sequences (sRBS) with a range of translation initiation strengths. **(D)** Large-scale transcriptomics data can be mined for promoter and RBS sequences that are active at similar levels across a broad range of strains.

Native machinery with a range of activities can be rapidly identified using single-cell transcriptomics (Figure 11A). Genes that are always active or active in a condition of interest can be identified, and their corresponding promoter and RBS sequences can be fused to a reporter like GFP for further characterization in the strain of interest (Li et al., 2015; Olson et al., 2015). This approach allows for the discovery of regulatory elements with the desired function, often without understanding the detailed mechanism by which they function.

An alternative approach is to use orthogonal biomolecular machinery that functions independently of the native host machinery (Figure 11B). The most commonly used orthogonal system is phage T7 RNA polymerase, which only transcribes genes downstream of a phage T7 promoter (Loeschcke et al., 2013). A T7 RNA polymerase circuit has been used to produce protein across three bacteria species (Kushwaha and Salis, 2015). The benefits of orthogonal translation have been demonstrated in *E. coli* (An and Chin, 2009; Darlington et al., 2018; Liu et al., 2018). However, they have not been implemented in environmentally-relevant strains. Instead, translation initiation is usually tuned by varying the RBS (Figure 11C), either leveraging native or engineered RBS (Salis et al., 2009).

A third alternative is to mine big data generated using metagenomics for regulatory elements that present similar activities across diverse organisms, which are called broad-host range elements (Figure 11D). With this approach, the intergenic regions found in diverse prokaryotic genomes can be screened for activities across different hosts (Johns et al., 2018). These efforts have found that some regulatory sequences are functional in many species while others are specific to one organism. This approach has also been useful for identifying broad-host genetic parts, such as transcriptional terminators (Amarelle et al., 2019), which are used to insulate modules from one another during circuit construction.

#### Environmental Microbe Successes

The genetic toolkits emerging for programming environmental microbes have been applied to a wide range of organisms. Some examples of newly-programmable microbes include: (1) predatory bacteria that live in soils and sediments (Dwidar and Yokobayashi, 2017), (2) pollutant-degrading bacteria found in soils, activated sludge, and sediments (Nikel et al., 2014; Tang et al., 2018), (3) gut bacteria from mice and bees (Mimee et al., 2015; Leonard et al., 2018; Riglar and Silver, 2018), (4) photosynthetic microorganisms from freshwater and marine settings (Markley et al., 2015; Crozet et al., 2018), (5) plant growth promoting rhizobacteria (Döhlemann et al., 2016; Guiziou et al., 2016; Zúñiga et al., 2018), (6) bacteria associated with marine phytoplankton and animals (Piekarski et al., 2009; Visick et al., 2018), (7) metal-reducing bacteria from lake sediment (Corts et al., 2019), and (8) bacteria from volcanic springs and hydrothermal vents (Reeve et al., 2016; Gilman et al., 2019).

### Cell-Free Reactions Enable Field Deployment

In contrast to living biosensors, cell-free reactions contain lyophilized biomolecules that simply require hydration to use. The result is a stable, portable sensor that can be field deployed for environmental measurements. Cell-free biosensors, also called transcription-translation or “TX-TL” reactions, sidestep many of the challenges with deploying living biosensors. They avoid challenges with biosensor viability under nutrient limiting or toxic conditions, remove the need for signals to pass through the cell membrane, and eliminate concerns about the spread of engineered organisms into the environment (Karig, 2017; Soltani et al., 2018). Cell-free biosensors can monitor environments across a range of scales (cm to km) and can be either qualitative (Gräwe et al., 2019; Verosloff et al., 2019; Jung et al., 2020; Thavarajah et al., 2020) or quantitative (Pardee et al., 2016) at the point of sampling. Finally, they avoid ethical issues of using living biosensors in the field.

#### Designing and Preparing Cell-Free Biosensors

**Figure 12:**
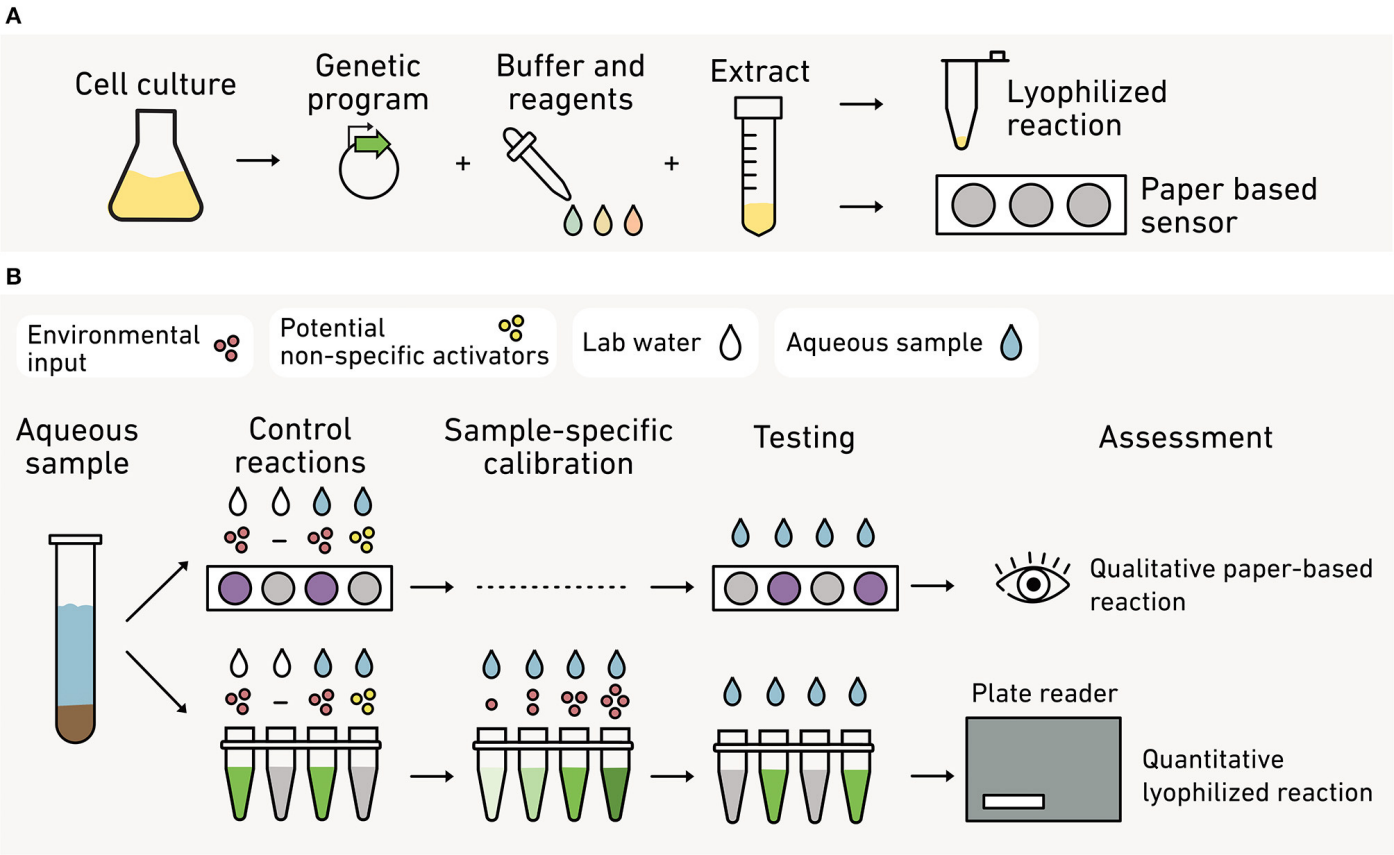
Cell-free systems as field-deployable biosensors. **(A)** To make these biosensors, cells are processed to create a lysate containing the necessary biomolecules for transcription and translation. DNA coding for the biosensor is added to the lysate as well as biochemical fuel (nucleotides and amino acids). The reaction can be lyophilized in tubes or freeze-dried on paper to create stable, portable biosensors. **(B)** With paper assays (*top*), qualitative visual outputs (*purple*) are used as a yes/no reporter of a given environmental conditions, such as the presence of an analyte (*red*) above a threshold concentration. Control reactions are used to determine if other analytes (*yellow*) non-specifically activate the sensor. With liquid assays (*bottom*), a lyophilized reaction is hydrated and analyzed with similar controls. In this approach, the output can be quantified against standards using a fluorimeter or spectrophotometer.

Cell extracts are first prepared from a microbial culture by lysing cells and removing membrane debris, leaving transcription and translational machinery necessary for protein synthesis (Figure 12A). A DNA program encoding the biosensing machinery is then added along with energy sources, nucleotides and amino acids for synthesizing this machinery, buffer, and salts (Levine et al., 2019; Silverman et al., 2019). Cell-free reactions can be used in liquid form or on paper for sensing. They can be flash frozen or freeze-dried to improve shelf life and portability (Pardee et al., 2014). With paper-based approaches, water is used to activate the biosensor in the field. Cell-free reactions are affordable to make or purchase, costing pennies per μL of TX-TL reaction (Levine et al., 2019).

Cell-free reactions are compatible with many sensing modules, signal processing circuits, and visual reporters (Soltani et al., 2018). Cell-free biosensors for monitoring environmental parameters have used transcription factors (Gräwe et al., 2019; McNerney et al., 2019; Jung et al., 2020) and RNA-based riboswitch sensors (Pardee et al., 2014, 2016; Verosloff et al., 2019; Thavarajah et al., 2020). Signal processing logic gates (Lehr et al., 2019) and feedback loops (Takahashi et al., 2015) have also been demonstrated in cell-free reactions. Finally, visual outputs can be used in cell-free biosensors for point-of-sampling diagnostics, including fluorescent proteins (Pardee et al., 2014, 2016; Gräwe et al., 2019; McNerney et al., 2019; Voyvodic et al., 2019; Thavarajah et al., 2020), fluorescent RNA (Jung et al., 2020; Thavarajah et al., 2020), luminescent proteins (Voyvodic et al., 2019), and pigment-producing enzymes (Pardee et al., 2016; McNerney et al., 2019; Verosloff et al., 2019; Thavarajah et al., 2020). The outputs from the cell-free biosensor can be qualitatively (Pardee et al., 2014, 2016; Gräwe et al., 2019; Verosloff et al., 2019; Thavarajah et al., 2020) or quantitatively visualized in the field (McNerney et al., 2019) (Figure 12B). Alternatively, samples can be transported back to the lab for quantitative analysis using a spectrophotometer and fluorimeter (Voyvodic et al., 2019; Jung et al., 2020).

#### Field Use of Cell-Free Biosensors

Lyophilized, cell-free biosensors are shelf-stable and portable, making them easy to store and transport to field sites (Jung et al., 2020; Thavarajah et al., 2020). Freeze-dried reactions are stable for up to 5 months under atmospheric conditions (Karig, 2017; Thavarajah et al., 2020) and for a year when stored under nitrogen (Pardee et al., 2014). Once an environmental sample is mixed with the cell-free reaction, output detection requires a few minutes (Pardee et al., 2014, 2016; Gräwe et al., 2019; Jung et al., 2020) to hours (Verosloff et al., 2019; Thavarajah et al., 2020). Cell-free biosensors that require incubation can be warmed by hand, minimizing the equipment needed for field use (Verosloff et al., 2019; Thavarajah et al., 2020).

To date, only a handful of cell-free reactions have been developed as biosensors for environmental applications. Jung et al. used cell-free reactions for field measurements of zinc and copper in municipal water samples in Paradise, CA following wildfire contamination (Jung et al., 2020). Thavarajah et al. also demonstrated on-site detection of fluoride in contaminated water in Cartago, Costa Rica (Thavarajah et al., 2020). Cell-free biosensors have also been used to detect quorum sensing molecules in sputum samples from patients with respiratory *Pseudomonas aeruginosa* infections (Wen et al., 2017), plant pathogens in tobacco leaf extract (Verosloff et al., 2019), cocaine in urine (Voyvodic et al., 2019), zinc in human serum (McNerney et al., 2019), and Zika virus in macaque plasma (Pardee et al., 2016).

Complex environmental samples may contain chemicals that sequester, destabilize, or inhibit the function of cell-free reactions (Wen et al., 2017; McNerney et al., 2019; Jung et al., 2020; Thavarajah et al., 2020). Serial dilutions of environmental samples can be used to overcome this challenge (Jung et al., 2020), and environment-specific calibration can help mitigate these matrix effects (McNerney et al., 2019).

## D. Current Engineering Needs

While the synthetic biology community has engineered an amazing range of biological modules over the past decade, these developments have largely been guided by biomedical applications. Many biological parts are not yet appropriate for studying environmental and Earth science questions. Key engineering problems remain unsolved, and application in environmental science will require attention to these challenges. To be truly useful for answering environmental questions, biosensors need to: (1) have sensing modules that are specific and sensitive to environmental inputs, (2) produce measurable outputs from within environmental samples that are easy to monitor, (3) function robustly over environmental study time scales, (4) use microbial strains of relevance to the processes being studied, and (5) be suitable for safe field applications.

### Inputs: Expand the Suite of Sensing Systems

There is currently a need to increase the number of sensing modules relevant to studying environmental processes and to expand the application of existing sensors to environmental questions. Biosensors that report on biogeochemical cycle intermediates (Table 1) could provide valuable information about the dynamics of intermediate fluxes or about cryptic cycling in environmental contexts. Sensors for redox-active chemicals could help link changes in dynamic environmental conditions to microbial community structure (Table 2), while sensors for enzymatic cofactors could differentiate the genetic potential of a microbe to perform a biochemical reaction from the expression of the enzyme and the enzyme's ability to perform that reaction, i.e., coordination of a cofactor essential to activity. Many environmental chemicals lack known sensor modules, highlighting engineering opportunities for developing sensors of relevance to Earth and environmental science questions. Specifically, there is a need for sensing modules that respond to intermediates in the carbon (e.g., methane, chitin degradation products, and cellulose degradation products), nitrogen (nitrous oxide), sulfur (elemental sulfur, sulfite, and sulfate), and phosphorous (nucleic acids) cycles. There is also a need for modules that respond to different oxidation states of metals essential to life (e.g., Mo^2+^, Mo^3+^, Mo^4+^, Mo^5+^, Mo^6+^), osmolytes (trehalose, dimethyl sulfoniopropionate, and taurine), signaling molecules (additional plant-microbe and microbe-microbe signals), critical materials (rare Earth elements), and environmental parameters (pH, electron donors, and electron acceptors). Finally, approaches for tuning input modules' specificity and sensitivity are needed to avoid signal interference from the complex ensemble of chemicals found in environmental samples.

### Outputs: Create More Environmentally-Compatible Reporters

The paucity of genetically-encoded outputs for *in situ* environmental studies has slowed biosensor applications in complex samples. Common visual reporters are difficult to monitor *in situ*, due to the high absorbance of light by soils and sediments. Enzymes that produce indicator gases show promise for overcoming some of these barriers (Cheng et al., 2016, 2018). However, there is still a need to develop additional non-invasive and stable outputs for environmental samples, such as a greater repertoire of gas channels for tracking cell growth and sensing in parallel. Ideally, these new indicator gases would be inert and non-toxic, and they would function under both anaerobic and aerobic conditions. For ease of engineering, indicator gases should be produced by a single enzyme using a common metabolite as a substrate. Gas vesicle outputs also show promise as reporters in some complex conditions, such as wastewater. These vesicles have been used as contrast agents in vertebrates for ultrasound and magnetic resonance imaging measurements (Lu et al., 2018; Farhadi et al., 2019) suggesting that could be used as outputs in environmental samples with further method development.

### Processing Modules: Record Complex Information *In situ*

Logic gates and cellular memory have the potential to greatly expand the types of environmental questions that biosensors can address. However, several engineering challenges remain to employing these components in environmental contexts. First, as the number of components in a genetic circuit increases, there is a greater potential for biological noise to propagate through the circuit and destabilize either the ON or OFF states (Pedraza and van Oudenaarden, 2005). For this reason, any biosensor employing processing modules needs to be thoroughly tested and refined in the target environment to ensure circuit stability. As described in section C, multiple types of biological memory have been employed to record the order and approximate time and duration of events. The long-term stability of these memory systems needs to be carefully evaluated and tuned before being applied in environmental contexts. Furthermore, most memory systems have been used in *E. coli*. Significant engineering opportunities exist to develop biological memory that functions across environmental microbes of relevance to diverse Earth and environmental sciences studies.

### Tuning: Design Sensors That Function Across Longer Time Scales

To function in a complex environmental sample, biosensors need to function on time-scales relevant to the processes under investigation. In some cases, this may require genetic programs that are stable for weeks to months, rather than the hours to days that are typical of most proof-of-concept biosensors. Tuning biosensors for long duration applications will involve methods for keeping the OFF states stable in the absence of inputs. Additionally, tuning will be required so that the ON state reports accurately and consistently across longer time scales following detection of an input. Genetic circuit stability for these longer durations will ultimately depend on the consistent function and stability of each system component, and it will require verification across diverse environmental microbes and samples.

### Chassis: Engineer More Environmentally-Relevant Microbes

High-performance conjugation systems hold promise for programming diverse environmental microbes (Brophy et al., 2018; Ronda et al., 2019; Wang et al., 2019), as do methods for targeted genome editing in communities (Rubin et al., 2020). However, conjugation systems have only been used to program microbiomes from a limited number of environmental settings. A current engineering opportunity is to apply broad host conjugation tools in diverse lake, marine, soil, and wastewater locations. An additional opportunity is to create a larger tool set of genetic parts that function across a wide range of environmental microbes. Currently, broad host range genetic parts are not sufficiently reliable across different environmental microbes, and they need to be screened on a case-by-case basis for each new application, which slows biosensor creation. Bioinformatic models can help screen and predict the activity of regulatory sequences, although data sets to train the predictive models are still limited for non-model organisms (Gilman et al., 2019). High throughput screening methods, such as massively parallel reporter assays, droplet microfluidics, and cell-free approaches, could be used to collect big data to guide the improvement of these models. These new genetic parts can then be incorporated into larger synthetic circuits in non-model organisms (Belliveau et al., 2018; Yim et al., 2019; Ding et al., 2020).

### Field Deployment: Engineer Safe, Reliable Biosensors

Cell-free sensors hold immediate promise for the safe use of biosensors in the field, and further engineering improvements could broaden their utility. The development of methods for using two component systems in cell-free systems would enable many new environmentally-relevant sensors, given the diversity of chemicals that can be sensed by members of this sensor module family. Environmental samples are chemically complex, and environmental compounds such as organic matter or charged ions have the potential to inactivate cell-free reactions by interfering with biomolecular components (McNerney et al., 2019; Jung et al., 2020). To enable easy and equipment-free measurements in the field, there is a need to create cell-free sensors that do not experience these limitations.

Whole cell biosensors also hold promise for applications in safe settings that prevent release into the environment, such as closed laboratory systems containing soil, sediment, and wastewater that can be autoclaved. To enable *in situ* applications, a range of biocontainment strategies have been developed. These include the creation of auxotrophic strains whose growth depends upon non-natural amino supplementation (Mandell et al., 2015; Simon and Ellington, 2016), the incorporation of kill switches that couple cell survival to the presence of a passcode chemical (Chan et al., 2016) or to environmental conditions (Stirling et al., 2017), and sequence entanglement techniques that prevent horizontal gene transfer with native microbiomes (Blazejewski et al., 2019). Another strategy involves embedding sensors in capsules or retrievable materials like alginate beads to reduce the risk of escape or genetic exchange with microbial communities (Shemer et al., 2020). While numerous biocontainment approaches have been described recently, more development and testing are needed with these different approaches to better understand their performance across diverse environmental microbes, communities, and materials. The utility of whole cell biosensors in the environment will only truly be realized when biocontainment strategies enable both robust functioning and the necessary safety.

## E. Ethical Considerations

Most of the immediate applications of synthetic biology in environmental sciences involve use of engineered organisms in the laboratory, where the ultimate fate of a biosensor is autoclave death. Laboratory studies with biosensors do not face ethical challenges as long as researchers adhere to modern microbiology laboratory standards. However, it is likely that researchers will eventually attempt to use synthetic microbes as detectors outside the laboratory and this raises a host of questions about risks associated with either accidental or intentional release of engineered organisms into the wild. The *National Institute of Health Guidelines for Research Involving Recombinant Or Synthetic Nucleic Acid Molecules* recommends a maximum escape frequency of synthetic *E. coli* corresponding to 10^−8^ cells (National Institutes of Health, 2019). Currently, there is a need to better understand how accidental release of other environmental microbes beyond this limit might persist in different terrestrial settings, since larger scale applications of synthetic microbes are being considered for bioproduction (Moe-Behrens et al., 2013; Wang and Zhang, 2019).

The misuse or poorly-planned use of synthetic organisms in the environment has the potential to damage the natural world. Every research project involving environmental synthetic organisms should include explicit consideration of ethics, with the extent of resources invested in ethical considerations increasing with the potential for environmental release. For example, experiments that use large populations of microbes that have a greater potential for release and environmental damage should be considering the local setting where release could occur and the implications of that environmental material for synthetic cell fitness. With laboratories strains of *E. coli*, the assumption has been that cell fitness will be low in such settings. However, when programming microbes native to an environmental niche, this may not be the case. These considerations should occur throughout the experimental timeline, and designs that violate either laws or ethical norms should not be developed under any circumstances. Because members of research groups planning an environmental release are unlikely to be unbiased in their evaluation, groups should engage scholars from outside their team with expertise in ethics of genetically modified organisms.

## Author Contributions

IDV, EF, PK, JS, CM, and LS conceptualized and wrote the manuscript, while IDV and EF synthesized the figures and table. All authors contributed to the article and approved the submitted version.

## Conflict of Interest

The authors declare that the research was conducted in the absence of any commercial or financial relationships that could be construed as a potential conflict of interest.
